# Approaching the Zero‐Power Operating Limit in a Self‐Coordinated Organic Protonic Synapse

**DOI:** 10.1002/advs.202305075

**Published:** 2023-10-23

**Authors:** Shuzhi Liu, Zhilong He, Bin Zhang, Xiaolong Zhong, Bingjie Guo, Weilin Chen, Hongxiao Duan, Yi Tong, Haidong He, Yu Chen, Gang Liu

**Affiliations:** ^1^ School of Chemistry and Chemical Engineering Shanghai Jiao Tong University Shanghai 200240 China; ^2^ School of Chemistry and Molecular Engineering East China University of Science and Technology Shanghai 200237 China; ^3^ Department of Micro/Nano Electronics School of Electronic Information and Electrical Engineering Shanghai Jiao Tong University Shanghai 200240 China; ^4^ Suzhou Laboratory Suzhou 215000 China; ^5^ Minhang Hospital Fudan University 170 Xinsong Road Shanghai 201199 China

**Keywords:** low power consumption, nonvolatile memory, organic protonic synapse, proton‐reservoir TPPS molecule

## Abstract

High‐performance artificial synapse with nonvolatile memory and low power consumption is a perfect candidate for brainoid intelligence. Unfortunately, due to the energy barrier paradox between ultra‐low power and nonvolatile modulation of device conductances, it is still a challenge at the moment to construct such ideal synapses. Herein, a proton‐reservoir type 4,4′,4″,4'''‐(Porphine‐5,10,15,20‐tetrayl) tetrakis (benzenesulfonic acid) (TPPS) molecule and fabricated organic protonic memristors with device width of 10 µm to 100 nm is synthesized. The occurrence of sequential proton migration and interfacial self‐coordinated doping will introduce new energy levels into the molecular bandgap, resulting in effective and nonvolatile modulation of device conductance over 64 continuous states with retention exceeding 30 min. The power consumptions of modulating and reading the device conductance approach the zero‐power operating limits, which range from 16.25 pW to 2.06 nW and 6.5 fW to 0.83 pW, respectively. Finally, a robust artificial synapse is successfully demonstrated, showing spiking‐rate‐dependent plasticity (SRDP) and spiking‐timing‐dependent plasticity (STDP) characteristics with ultra‐low power of 0.66 to 0.82 pW, as well as 100 long‐term depression (LTD)/potentiation (LTP) cycles with 0.14%/0.30% weight variations.

## Graphical abstract

A proton‐reservoir type TPPS molecule is designed and synthesized to deliver organic synapses exhibiting 64‐state conductance modulation characteristic with ultra‐low power consumption of 6.5 fW to 0.83 pW and retention over 30 min. The critical contradiction between low‐power operation and long‐term stability of organic synapses is solved with the present TPPS molecule‐based devices.

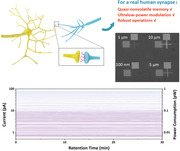

## 1 Introduction

Molecular neuromorphic device is an important part of constituting bio‐inspired electronics.^[^ ^1^, ^2^ ^]^ It utilizes the conductance evolution dynamics of organic molecules to emulate the weight modulation characteristics, as well as the neural signal recording, processing and transmitting functions of biological synapses, demonstrating great application potential in intelligent perception, advanced storage, logic, and brainoid computing areas.^[^ ^3^, ^4^, ^5^ ^]^ Being different from silicon transistors that encode “0” and “1” based on the electric field effect‐driven volatile switching of the device conductances, biological neural system relies on chemical species such as ions and neurotransmitters to nonvolatilly control the synaptic strength that connects neurons.^[^ ^6^, ^7^ ^]^ As such, the complex tasks of learning, memorizing, recognition, decision, and etc., can be executed in an energy‐efficient manner in the giant neural network through in situ storage and processing of the biological signals. In order to emulate the neuromorphic signal processing capability of biological synapses, the conductance tuning behavior of organic device should also exhibit low‐power and nonvolatile features.

**Figure 1:**
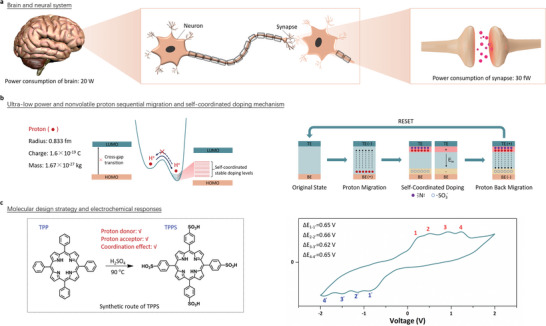
a) Schematic illustration of the brain and neural system containing neurons and synapses. b) Energy band diagram and working mechanisms based on proton sequential migration and self‐coordinated doping effects. c) Molecular design strategy, synthetic route and electrochemical response of the proton‐reservoir type molecule TPPS.

As a robust biological computer, the power consumption of brain and individual synapses reaches as tiny as 20 W and tens of femtowatt, respectively (**Figure** **1**a).^[^ ^8^ ^]^ The realization of high‐speed and low‐power conductance switching in molecular devices requires to effectively lower the energy barriers for both the transition of materials’ intrinsic states and cross‐band transition of charge carriers. Nevertheless, lowering the transition barrier will also elevate the possibility of spontaneous relaxation of the materials’ state, which in turn drives the molecular system into a metastable circumstance with shorter retention performance. For instance, charge transfer (CT) strategy adopted from organic light‐emitting diodes and solar cells has been employed to tune conductance of molecular neuromorphic devices.^[^ ^9^, ^10^, ^11^ ^]^ Upon narrowing the bandgap of organic semiconductor through molecular orbital hybridization, electric field‐induced charge transfer between donor and acceptor moieties can inject electrons from HOMO into LUMO of the macromolecules easily, resulting in half‐filled molecular orbits and highly conductive CT complex that is essentially semi‐separated electron‐hole pairs (Figure S1a, Supporting Information). Sub‐10 nanosecond generation of currents and photocurrents in organic device can be achieved by such CT mechanism.^[^ ^12^ ^]^ Due to the lack of proper mechanism to stabilize the activated CT state, however, spontaneous back‐migration and recombination of electrons and holes upon removal of external electric field will inevitably lead to fast device relaxation to pristine low conductance state. This is in fundamental contradiction with the nonvolatile synaptic weight tuning demands for computing‐in‐memory (CIM) paradigm and is the major challenge for early‐stage investigation of molecular neuromorphic devices.

Pioneering works also attempt to achieve continuous modulation of organic device conductance by forming local conductive filaments (CFs) through the injection, migration and redox of metal cations from the chemically active electrode.^[^ ^13^, ^14^, ^15^, ^16^ ^]^ In order to realize the nonvolatile tuning characteristics, high electric field is usually used to form thick metallic CFs that do not dissolve spontaneously under concentration gradient with the surrounding organic matrix. It is noteworthy that formation of such thick filaments not only involves high electric field to inject sufficient amount of metal ions from electrode into organic switching layer, but also leads to high current flowing through the highly metallically conductive CFs, which restrict these conductive‐bridge type devices from large scale integration and applications (Figure S1b, Supporting Information). Deeply understanding the key factors that influence transport properties of organic devices, as well as designing molecules that possess high‐speed and stable responses, deserve devotion of great efforts to compromise the paradox of ultra‐low power and nonvolatile conductance modulation on material and mechanism levels. It is of critical importance for the implementation of high energy‐efficiency and reliable molecular neuromorphic devices and systems.

Herein, we design a proton‐reservoir type organic molecule, 4,4′,4″,4'''‐(Porphine‐5,10,15, 20‐tetrayl) tetrakis (benzenesulfonic acid) (TPPS), and successfully fabricated an artificial synapse that possesses simultaneous ultra‐low power and nonvolatile characteristics via proton migration and self‐coordinated doping effect. By incorporating proton‐donating sulfonic acid and proton‐accepting amino units into the planar porphyrins molecules as reservoir, sequential migration and doping of protons can effectively tune local region composition and energy bandgap of the molecular thin film with a series of intermediate states. Self‐coordination between the migrated protons and lone‐pair electrons of amino groups adjacent to the counter electrode then stabilize the activated state molecules effectively, leading to 64 levels continuous modulation of device currents between 650.4 fA and 82.5 pA with quasi‐nonvolatile retention over 30 min in Au/TPPS/Au organic protonic memristor (OPM). The power consumption of their single bit conductance modulating and reading operations fall into low level of 16.25 pW to 2.06 nW and 6.5 fW to 0.83 pW, respectively, which already meets the criterion of biological synapses. Such approaching‐zero‐power‐limit behavior of the proton‐reservoir type organic synapse devices offers important solution for solving the ultrafast and nonvolatile conductance modulation paradox. Finally, we showcase ultra‐low power spike‐related plasticities with the present OPM, wherein 100 successive long‐term depression (LTD) and potentiation (LTP) cycles with 0.14% and 0.30% plasticity variations, respectively, again confirm the robustness of the self‐coordinated organic proton synapse.

## 2 Results and Discussion

### 2.1 Design Essence of the Self‐Coordinated Molecule and Organic Protonic Memristors

Proton is the smallest ion in the world. Its charge‐to‐mass ratio (1.6 × 10^−19^ C/1.67 × 10^−27^ kg) is far larger than that of the metal cations (e.g., 1.6 × 10^−19^ C/1.79 × 10^−25^ kg for Ag^+^), therefore enabling facile migration under low electric field in organic materials. In the meanwhile, the effective mass of proton (1.67 × 10^−27^ kg) is over three orders of magnitude larger than that of electrons (9.11 × 10^−31^ kg), which can significantly attenuate the possibility and speed of spontaneous backward migration upon removal of external electric field. Therefore, proton is considered as a promising carrier for ultra‐low power and nonvolatile conductance modulation in organic devices.^[^ ^17^ ^]^ In this contribution, we design a reservoir‐type organic molecule TPPS that simultaneously consists of proton donating sulfonic acid and accepting amino chromophores in the chemical structure. In TPPS molecules and OPM devices, protons of sulfonic acid can migrate toward counter electrode under low electric field, introducing intermediate states into the energy bandgap and establishing build‐in field across the organic thin film (Figure 1b). Consequently, inter‐state transition and transport of charge carriers can be facilitated vastly, resulting in continuous potentiation of device conductance in a low‐power manner. The migrated protons will be grabbed by amino units of the TPPS molecules near the counter electrode via coordination between H^+^ empty 1s orbit and unbonded electron pairs of amino nitrogen atoms, impeding them from concentration gradient driven drift to the original position. This procedure is similar to the neurotransmitter release, transport, and binding with post‐synapse receptors that nonvolatilly mediate synaptic connection strength in biological neural system,^[^ ^18^, ^19^ ^]^ which in turn ensures long‐term stability of organic devices. According to the above concept, we successfully synthesized TPPS molecules through sulfonation reaction of 5,10,15,20‐tetraphenylporphyrin (TPP), as shown in the left panel of Figure 1c and confirmed by nuclear magnetic resonance (NMR), matrix‐assisted laser desorption/ionization time of flight mass spectrometry (MALDI TOF‐MS) and Fourier transformed infrared (FT‐IR) spectra. The detailed information of synthesis, electrochemical, optical, and structural characterizations of TPPS are summarized in Figures S2–S7 (Supporting Information). It is noteworthy that the cyclic voltammetry spectrum of TPPS film drop‐casted on a platinum disk working electrode exhibits quadruple peaks in either the oxidization and reduction branches (right panel of Figure 1c). The oxidation peaks locate at the potentials of 0.25, 0.50, 0.90, and 1.24 V, while the reduction peak potentials are −0.90, −1.16, −1.52, and −1.89 V, respectively. For each pair of the oxidization‐reduction peaks of the quadruple redox characteristics, the differences in the absolute values of the oxidization and reduction potentials are almost constant of 0.62–0.66 V. It coincides with the sequential release and retrieving of the four protons from each TPPS molecules.

### 2.2 Electrical Performance of the OPM

**Figure 2:**
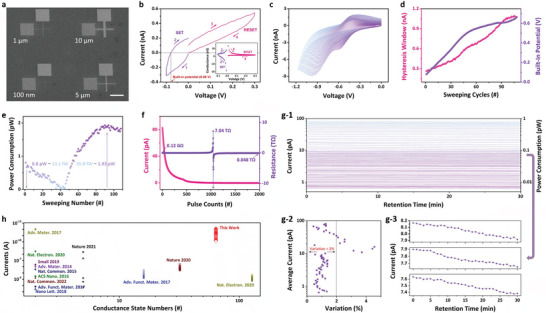
a) Scanning electron microscopic image of the cross‐point structure Au/TPPS/Au OPM devices with electrode linewidth of 10 µm, 5 µm, 1 µm, and 100 nm, respectively. Bar scale: 60 µm. b) Current–voltage characteristics of the OPM device obtained by voltage sweepings from 0 to −0.1 to 0 V and from 0 to 0.3 to 0 V, respectively. Inset replots the conductance–voltage curve of the device during the same sweeping periods. c) Current–voltage characteristics of the OPM device obtained during consecutive negatively biased voltage sweepings with the stopping voltages increasing from −0.1 to −1.17 V with a ramping step of −0.01 V. Evolution of d) the memory hysteresis windows of the OPM device and built‐in potentials established across the TPPS film, as well as e) the power consumptions to read the device during the above negatively biased voltage sweepings. f) Evolution of the device currents and resistances as a function of the applied pulse numbers during the modulation with positive modulation pulse (amplitude: 0.5 V; width: 10 µs; interval: 140 µs) and a read voltage of −0.01 V. g‐1) Retention performance of 64 continuous conductance states obtained during the pulse modulation process. The device current was read using a voltage pulse with the amplitude of −0.01 V and a width of 10 µs. g‐2) summarizes the variations of the 64 state device currents recorded during the sampling period of 30 min. g‐3) displays the retention performance of 3 conductance states with middle device currents among 64 conductance states. These neighboring conductance states are inter‐distinguishable for at least 30 min. h) Comparison of device currents and number of conductances between the TPPS based OPM in this work and other memristor synapses reported in the literatures.

Based on the above molecular design and mechanism analysis, we fabricated OPM devices with the structure of Au/TPPS/Au, organic film thickness of 18 nm, and different electrode linewidth of 10 µm, 5 µm, 1 µm, and 100 nm, respectively ( **Figure** **2**a; Figure S10, Supporting Information). Detailed information on device fabrication is summarized in the Experimental Section. Before fabricating the OPM devices, the compatibility of TPPS powders and thin films with the electron‐beam lithography process was double‐confirmed through solubility and spectroscopic tests (Section S7, Supporting Information). In all electrical measurements of this work, the biased voltages were applied onto the top electrodes while the bottom electrodes were always grounded. Other than specifically mentioned, the current–voltage (*I*–*V*) characteristics and electrical performance discussed below are assessed on 10 µm OPM devices. Since the device has a symmetric structure of Au/TPPS/Au, the polarity of the applied voltage sweeps will not influence the shape of the resultant current–voltage or conductance–voltage characteristics significantly. The only difference would be that a mirror image of the *I*–*V*/*G*–*V* curves will be obtained when the polarity and applying sequence of the applied voltage sweeps are reversed. In this work, we first applied negatively biased voltage sweepings onto the device to potentiate its conductance, therefore, we call the negative sweep SET and the positive sweep RESET processes. As shown in Figure 2b and Figure S11 (Supporting Information), when a voltage sweep between 0 and −0.1 V is applied, the device current rises from −80.64 pA at −0.01 V to −0.31 nA at −0.1 V. Interestingly, back scanning reveals that the polarity of device current gets reversed, becoming 0.18 nA when read again at −0.01 V, suggesting that a built‐in electric potential has been established across the organic film. The nonlinear evolution of the TPPS device currents during forward and backward sweepings, in particular the hysteresis in the current–voltage curve, indicates the occurrence of conductance switching in the molecular layer. We also replot Figure 2b in the form of conductance–voltage characteristic. As shown in the inset, the conductance of the TPPS device changes obviously during dc voltage sweeping.

Further dual directional voltage sweepings with the stopping voltage increasing from −0.1 to −1.17 V with a ramping step of −0.01 V result in 108 continuous modulation cycles of the device currents and conductances, as shown in Figure 2c and Figure S12 (Supporting Information), respectively. As the stopping voltage increases, the recorded current–voltage hysteresis windows become widened consecutively. By defining as the differences of device currents recorded at −0.01 V during the forward and backward scans, the hysteresis windows increase from 0.27 to 1.07 nA by four times (Figure 2d). The built‐in potential, which can be read as the nominal voltage when the device currents decay to zero during backward scan, also increases from 0.08 to 0.67 V monotonically. The built‐in potential should be related to directional migration and accumulation of protons toward the top electrode in negatively biased voltage sweepings, producing an additional downward pointing electric field across the organic thin film. As proton migration continues with the applied external electric field, the built‐in potential increases accordingly.

Since neuromorphic computing using artificial synaptic devices is mainly based on multiply‐and‐accumulation (MAC) that can be done in a single read‐out operation, we calculate the power consumption of reading the present TPPS device to assess its low‐power performance. Herein, the power consumption of reading the OPM is calculated by multiplying the absolute values of the read voltage (−0.01 V) and device currents (read at −0.01 V) during continuous sweeping modulation. Note that the net electric field addressing on TPPS thin film is an overall result of external reading voltage and built‐in potential with reverse polarity. Along with the increase of built‐in potential, the net electric field and read current of the organic device both decreases, giving rise to a decreasing power consumption ranging from 0.81 pW for sweep with the stopping voltage of −0.1 V to 13.1 fW for sweep with the stopping voltage of −0.51 V, respectively (Figure 2e). The built‐in potential equals external reading voltage in the 42nd sweep, which results in near‐zero‐power operation characteristics of the device. Afterward, the amplitude of built‐in potential exceeds that of external reading electric field, leading to a gradually increased device current with reversed polarity and an increasing power consumption from 35.8 fW to 1.93 pW, respectively. A negative differential resistance and power consumption region can also be observed for sweeps with the stopping voltages of −0.92–−1.17 V. It may also be understood by built‐in potential dominated transport of charge carriers upon proton migration and accumulation and will be discussed in later section.

The reset process of the OPM can be achieved with a positive voltage sweeping from 0 to 0.3 V, during which the migrated protons fully return to their initial sites and the device is programmed completely to the pristine low conductance state (Figure 2b). In order to realize consecutive modulation of the device conductance, we utilize operations of positive pulse (amplitude: 0.5 V; width: 10 µs; interval: 140 µs) and a subsequent 100 ms read pulse of −0.01 V to replace the sweeping operation. The application of these positive voltage pulses will push the migrated protons from the top electrode/organic layer interfacial region to their initial position near the bottom electrode. Upon the recombination of the protons with the negatively charged sulfonic anions, the additional doping energy level introduced in TPPS's bandgap among ion migration will be ceased. Simultaneously, the built‐in potential also decreases along this procedure. As depicted in Figure 2f, the device current decreases from 82.86 pA to 1.40 fA for the first 1046 pulses consequently. As the process continues, all the migrated protons return to their original position and recombine with the sulfonic anions. Afterward, the protons residing on the TPPS molecules near the top electrode start to migrate toward the bottom electrode. Then these protons will form an up‐pointing built‐in potential with the sulfonic anions near the top electrode. It counter‐balances the positive voltage applied across the organic thin film and further decreases the device current. When the amplitudes of the built‐in potential exceed that of the applied voltage, the polarity of the overall bias acting on the TPPS layer and thus the device current get reversed. In the following 954 positive pulses, the negative device current gradually increases to −0.21 pA. Please also see the following section for detailed working mechanism of the organic synapse. During this process, the resistance of the device increases from 0.12 × 10^9^ to 7.04 × 10^12^ Ω in the first stage, then decreases to 0.48 × 10^11^ Ω with reverse polarity in the second stage. The power consumption of modulating the TPPS device conductance during pulse‐mode operation can be estimated with the formular *P* = |*I*| ×  *V* =  |*V*/*R*|  ×  *V* =  *V* ^2^/*R*, where *P* is the power consumption of modulating the OPM, *V* is the amplitude of the modulating voltage pulse (0.5 V), *I* and *R* are the device current, and resistance obtained during modulation. The power consumptions of modulating the OPM in the above pulse‐mode process vary between 2.08 nW and 35.5 fW in the first stage, as well as 35.5 fW and 5.21 pW in the second stage, respectively.

We further monitor the retention performance of 64 linear continuous conductance states that are obtained during pulse modulation, with device current and read power consumption ranging from 650.4 fA to 82.5 pA and 6.5 fW to 0.83 pW, respectively. Herein, a voltage pulse with the amplitude of −0.01 V and width of 100 ms was used to read the device current periodically. The modulating power consumption of the 64 conductance states are 16.25 pW to 2.06 nW for the TPPS device, according to the formular *P* = *V* ^2^/*R* = *V* ^2^/|*V*′/*I*′| = *V* ^2^|*I*′/*V*′|, where *V* is the amplitude of the modulating voltage pulse (0.5 V), while *R*, *V’*, and *I’* are the device resistance, reading voltage (−0.01 V) and device current obtained during 64 state modulation. Figure 2g reveals that all 64 states can be held and clearly distinguished for at least 30 min, showing a quasi‐nonvolatile memory characteristic. Only tiny variations of 0.44% to 4.56% are observed for the 64‐state device currents during the sampling period of 30 min, according to the following Equation (1):

(1) $$\mathrm{Variation}\left(\%\right)=\delta /\mu \times 100\%$$

where *δ* and *µ* is the standard deviation and average value of the device currents in each state. Hence, the contradiction between nonvolatile and low‐power operation can be resolved in the present TPPS OPM devices. Although these conductance states become inter‐crossed afterward, the 30 min retention capability already approaches short‐term memory (STM) level of biological neural systems.^[^ ^20^ ^]^ Such self‐decaying memory in biological brains also helps to lower the occupancy rate of synapses. After completing the demanded neural tasks, synaptic memory decays to resting level rapidly, thereby awaiting other tasks distributed by the brain. This can not only reduce the power consumption and workloads of neural system significantly, but may also extend its lifetime effectively.

When the OPM is swept from −1.0 to −5.0 V and from 0.1 to 1.0 V, similar continuous modulation of device currents is also observed (Figures S13–S15, Supporting Information). In comparison with the literatures that reported low‐power organic material, 2D material, as well as valance‐change‐memory (VCM) and electrochemical‐metallization (ECM) type metal oxide based devices,^[^ ^21^, ^22^, ^23^, ^24^, ^25^, ^26^, ^27^, ^28^, ^29^, ^30^, ^31^, ^32^, ^33^ ^]^ the TPPS self‐coordinated organic protonic synapse demonstrated in this work exhibits the lowest device currents of 650.4 fA to 82.5 pA and approaching‐zero read‐power‐consumption (6.5 fW to 0.83 pW) operation characteristics (Figure 2h). Together with the quasi‐nonvolatile memory capability of at least 30 min, the TPPS‐based devices are promising candidates for high energy‐efficiency and reliable molecular neuromorphic devices and systems. Lower‐power conductance modulation can be observed in cross‐point structure TPPS synapses with metal electrode linewidths of 5 µm, 1 µm and 100 nm, respectively (Figure S16, Supporting Information). The linear dependence of device currents with device areas indicates that the conductance modulation of the TPPS based OPM devices occurs as an interfacial phenomenon.

### 2.3 Interfacial Switching Mechanism of the OPM

To investigate the conductance switching mechanism of the TPPS‐based OPM, we further look into details of the current–voltage characteristics displayed in Figure 2c and Figure S13a (Supporting Information), and replot them in Figure S17 (Supporting Information). As shown, the current–voltage characteristics of the TPPS device can be divided into several slow‐current‐increasing, fast‐current‐increasing and negative differential resistance (NDR) regions. When the OPM device is swept with stopping voltage increasing from −0.10 to −0.33 V, the first batch proton cations of the TPPS molecules become excited and start to migrate across the organic film toward the top electrode, leaving behind negatively charged TPPS‐1H species containing immobile sulfonic anions. When these protons reach the top electrode/organic film interface, their empty 1s orbits will coordinate with the unbonded electron pairs of amino nitrogen atoms on the interfacial neutral TPPS molecules. Such self‐coordination will stabilize the migrated protons, the accumulation of which will not only lead to quasi‐nonvolatile conductance characteristics of the OPM device, but also result in a downward pointing built‐in potential with the sulfonic anions left near the organic film/bottom electrode (right panel of Figure 1b). The polarity of built‐in potential is opposite to that of external electric field. It hinders transport of charge carriers across the organic film, leading to slowly increasing current in the first part of the *I*–*V* curve. On the other hand, the doping energy levels introduced into the initial bandgap of the TPPS molecule through ion migration facilitate intramolecular charge carrier transition and long‐range transport across the organic film under external bias (left panel of Figure 1b). When the influence of the applied electric field on charge carrier transport overwhelms that of the built‐in potential, with the stopping voltages of the sweeps varying between −0.33 and −0.56 V, a fast increase in device current is observed.

**Figure 3:**
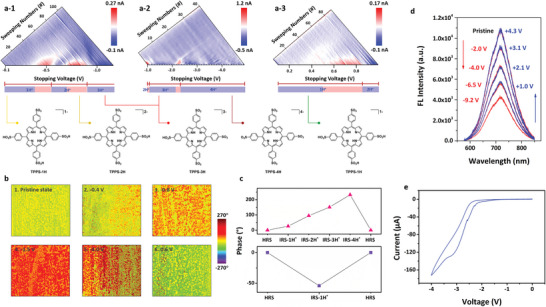
a) Pseudo‐color plots of the current changes between the adjacent current–voltage curves shown in Figure 2c and Figures S13a and S14a (Supporting Information). The lower panel display sequential removal of protons from TPPS upon excitation and migration, resulting in negatively charged TPPS‐1H, TPPS‐2H, TPPS‐3H and TPPS‐4H species. b) PFM images showing polarization characteristics of the TPPS film in a 1 µm × 1 µm scanning area under negative b‐1 to b‐5) and positive b‐6) voltage biases, respectively. c) Evolution of the PFM phases in the pristine HRS and low resistance states with the migration of different numbers of protons (IRS‐1H^+^, IRS‐2H^+^, IRS‐3H^+^, IRS‐4H^+^). d) Evolution of the TPPS fluorescence intensity with the applied voltage in the lateral Au/TPPS/Au devices. e) Current–voltage hysteresis of the lateral Au/TPPS/Au device recorded during dc voltage sweeping between 0 and −4 V.

In order to achieve a concise visualization of the above mention process, we make a device current subtraction between the *I*–*V* curves of the adjacent sweeping operations in Figure 2c and redraw it in **Figure** **3**a‐1. As shown, when the TPPS device is swept in the first 47 cycles with stopping voltage increasing from −0.10 to −0.56 V, an obvious increase in device current of 188.2 pA appears in −0.33 to −0.56 V region of the pseudo‐color current change plot. The blue signals in the −0.10 to −0.33 V region, nevertheless, reveals that current modulation is minor when the built‐in potential dominates the charge carrier transport procedure. When the stopping voltages increase from −0.56 to −0.92 V and from −0.92 to −2.60 V, the second and third batches of protons migrate toward the top electrode, get accumulated and generate TPPS‐2H and TPPS‐3H species near the bottom electrode, consequently. Arising from the compromise between the effect of the built‐in potential and external sweeping voltages, both slow‐current‐increasing and fast‐current‐increasing phenomena are demonstrated in Figure S17 (Supporting Information). Note that NDR is also shown when the stopping voltages of negatively biased sweeps vary between −0.92 and −1.17 V. It can be explained by the electrostatic repulsion generated by the protons accumulated near the top electrode/organic film interface, which may impede further transport of holes toward them. As depicted in Figure 3a‐1,a‐2, the maximum current increase resultant from the migration of the second and third batch protons are 0.27 and 0.69 nA, respectively. When the fourth and last batch protons of the TPPS molecules migrate during sweeps with stopping voltages increasing from −2.6 to −5.0 V, the absolute value of the device current exhibits a gradual increase from 11.44 to 11.96 nA (Figures S13a and S17, Supporting Information). Similar conductance modulation with built‐in potential dominated slow‐current‐increasing and external electric field dominated fast‐current‐increasing regions is also observed in positively biased voltage sweepings (Figure 3a‐3; Figure S14a, Supporting Information).

We further conducted molecular simulation to confirm the sequence of proton migration from the TPPS molecules, and correlate it with the monitored conductance modulation characteristics of the OPM devices. As shown in the left panel of Figure 1c and lower panel of Figure 3a, each TPPS molecule contains 4 protons. Excitation and migration of the first proton from the TPPS molecule will result in a negatively charged TPPS‐1H species. In order to reach the most stable configuration with lowest energy, the sulfonic groups will rotate around the S─C bonds that connect themselves to the porphyrin framework, leading to decreasing dihedral angles from 67.49°, 68.67°, 68.11°, and 68.53° for the neutral TPPS molecule to 65.40°, 65.25°, 61.05°, and 67.21° for TPPS‐1H between sulfonic groups and porphyrin framework (Figure S18, Supporting Information). Excitation and migration of the second proton have two possibilities, which are from the sulfonic acid groups at the ortho‐ and para‐positions of the first proton and sulfonic acid group, respectively. Removing the para‐position proton from TPPS‐1H will give rise to TPPS‐2H with the farthest distance and smallest intra‐molecular repulsion between the resultant negative charges, therefore, most stable configuration with enhanced co‐planarity of the divalent molecule. This is consistent with the further decreasing dihedral angles between the sulfonic groups and porphyrin framework, which are 64.27°, 63.91°, 64.26°, and 63.91°, respectively. Further excitation of the third and fourth protons from the charged TPPS molecules have no preferences, as they are equivalent in terms of intra‐molecular positions. Such sequential excitation and migration of protons are in good agreements with the near‐constant potential differences of the quadruple oxidization and reduction pairs depicted in the right panel of Figure 1c.

Piezo force microscopic (PFM) measurements were also performed to better understand the conductance modulation mechanism of the TPPS based OPM devices. Being similar to the conductive atomic force microscopy (C‐AFM) observation, the PFM measurements use Kelvin probe as movable top electrode to form a Pt/TPPS/Au device with TPPS film deposited on Au coated SiO_2_/Si substrate. As displayed in Figure 3b‐1, the pristine TPPS film exhibits almost no polarization characteristics with PFM phase signal read at random sampling point to be as small as 0.21°. When a −0.4 V voltage was scanned over the 1 µm × 1 µm region through a point‐by‐point manner with the Pt Kelvin tip, obvious polarization of the organic film can be observed. In accordance with the upward migration of the first batch protons and accumulation of positive charges on sample surface, the phase value becomes 26.4° (Figure 3b‐2). Further increasing the scanning voltage from −0.8 to −1.5, and −3.0 V significantly promotes the migration and accumulation of the second, third and fourth batches of protons on the sample surface, giving rise to stronger polarization and larger phase values of 94.8°, 150.9°, and 232.2°, respectively (Figure 3b‐3,b‐5). Interestingly, the polarization occurs featurelessly over the entire TPPS film, indicating that the electric field‐induced migration and self‐coordination of protons are interfacial effects. When we reverse the polarity of the scanning voltage to induce downward migration of protons, opposite polarization across the organic film can be demonstrated (Figure 3b‐6). The continuous evolution of the TPPS film polarization characteristics is also summarized in Figure 3c. As the OPM device transits between the initial high resistance state (HRS) and low resistance states with the migration of different numbers of protons (IRS‐1H^+^, IRS‐2H^+^, IRS‐3H^+^, IRS‐4H^+^), the PFM phase signal of the TPPS film changes continuously and reversibly. This is consistent with the continuous and bistable modulation of device conductance in negatively biased voltage sweeps.

In order to validate the occurrence of proton migration in TPPS thin film and devices under external bias, electrochemical fluorescence measurement was conducted in situ to monitor both the device current response and fluorescence characteristics of the porphyrin skeleton. A lateral structured Au/TPPS/Au device was used instead of the sandwich structure devices, wherein the TPPS switching layer can be directly exposed to optical illumination and spectroscopic measurement (Figure S19a, Supporting Information). Upon absorbing sufficient energy through optical illumination, the ground‐state electrons of an organic material can be stimulated into the excited state. When these electrons return to ground state, the absorbed optical energy can be released in the form of fluorescence emission. In case of TPPS molecules, as the electron cloud resides on the porphyrin skeleton in both the ground and excited state (Figure S19b, Supporting Information), fluorescence should therefore occur on the porphyrin fluorophore. With the migration and removal of protons from TPPS molecule, an obvious shift of the electron cloud from the central porphyrin skeleton to the peripheral strong electron‐withdrawing sulfonic moiety occurs through the intramolecular charge transfer (ICT) interaction (Figure S19c, Supporting Information). It is expected that the fluorescence of the porphyrin fluorophore will be significantly hindered as a result.^[^ ^34^ ^]^

In good agreement with that of Figure S4 (Supporting Information), TPPS thin film in the lateral structure device shows strong emission intensity of 10.73 × 10^3^ a.u. at the wavelength of 717 nm, when excited by visible illumination of 532 nm (Figure 3d). When a −2.0 V voltage stimulus is applied across the parallel Au electrodes, the fluorescence intensity recorded near the anode decreases obviously to 8.99 × 10^3^ a.u., which can be explained in terms of shift of electron cloud from the porphyrin skeleton to the sulfonic moiety upon proton migration and ICT interaction. Further increasing the voltage from −4.0 to −6.5, and −9.2 V continuously attenuates the fluorescence intensity monitored near the anode, from 7.28 × 10^3^ a.u. to 5.47 × 10^3^ a.u. and 4.19 × 10^3^ a.u., respectively. Coincidently, hysteresis in the current–voltage characteristics and thus conductance switching of the lateral Au/TPPS/Au device are also observed (Figure 3e). When the polarity of the applied voltage stimulus is reversed with the intensity increasing from 2.1 to 3.1 and 4.3 V, the fluorescence intensity of the TPPS thin film at the same sampling position recovers from 7.15 × 10^3^ a.u. to 10.63 × 10^3^ a.u. that is in close proximity to the initial value. Therefore, the occurrence of proton migration in TPPS thin film and its correlation with the conductance switching characteristics of the OPM device can be confidently verified through the above in situ electrochemical fluorescence and transport property measurements.

### 2.4 An Approaching‐Zero‐Power Organic Protonic Synapse

**Figure 4:**
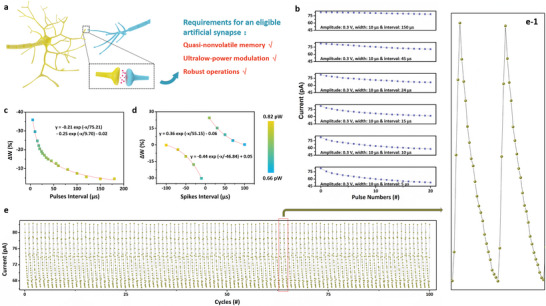
a) Schematic diagram of a biological synapse with quasi‐nonvolatile memory, ultralow‐power modulation and robust operation characteristics. b) Evolution of TPPS device current during long‐term depression as a function of the pulse interval varying between 150 and 5 µs. The amplitude and width of the modulating voltage pulses are identical, which are 0.3 V and 10 µs, respectively. The ultra‐low power c) spike‐rate‐dependent plasticity and d) spike‐timing‐dependent plasticity characteristics of the TPPS based OPM synapse. e,e‐1) LTD and LTP endurance performance of the TPPS synapse evaluated in 100 continuous modulation cycles. In each of the modulation cycles, 20 voltage pulses with the amplitude of 0.3 V, width of 10 µs and interval of 24 µs to achieve LTD, and subsequent 4 voltage pulses with the amplitude of −0.3 V, width of 10 µs and interval of 5 µs to achieve LTP are applied onto the device consecutively. All the currents were read using a voltage pulse with the amplitude of −0.01 V and width of 100 ms.

The operation of the human brain, with inherent combination of information storage and processing capabilities, depends on the unique synaptic activities. This enables the brain to perform a wide range of complex cognitive and recognition functions, including memory management, linguistic comprehension, target recognition, abstract reasoning, and etc. Although human brain is a complicated supersystem containing ≈10^11^ neurons and 10^15^ synapses, it consumes only ≈ 20 W power, comparable with that of an incandescent lamp. Considering a normal person with brain utilization rate of 5%, the power consumption of individual synapses can be roughly estimated as tens of fW. Besides, the human brain can work robustly for tens of years, during which some unimportant memory will be gradually forgotten with a quasi‐nonvolatile decay. As discussed previously, the self‐decaying memory can release synapses for executing other future neural tasks, thus increasing both the efficiency and lifetime of the brains. Although many efforts have been provided to imitate brain functions, achieving biological power consumption, quasi‐nonvolatile memory and robust operations simultaneously on individual synapse level is still challenging (**Figure** **4**a).

With the TPPS based OPM devices that exhibit both low‐power and non‐volatile memory characteristics, we further assess its capability of acting as an artificial synapse. Basically, the increase of device current upon proton migration can be deemed as a LTP plasticity, while the pulse‐induced decrease of device current is similar to the LTD plasticity. As depicted in Figure 4b, when a series voltage pulses with the amplitudes of 0.3 V and width of 10 µs are applied onto the OPM, the device current becomes attenuated gradually. As the pulse interval drops from 150, 45, 24, 15, 10, to 5 µs, an obviously enhanced depression of device currents is demonstrated. After the application of 6 pulse trains with the above intervals, the final device currents read at −0.01 V become 78.49, 70.64, 66.39, 61.86, 57.86, and 52.68 pA, respectively. Spiking‐rate‐dependent plasticity (SRDP) with approaching‐zero‐power characteristics can thus be simulated by the present TPPS device (Figure 4c) and fitted with the following double exponential function:

(2) $$\Delta \omega =C_{1}\;\exp\left(-t/\tau_{1}\right)+C_{2}\;\exp\left(-t/\tau_{2}\right)+b$$

where *Δω* is the change rate of synapse weight (device conductance) after the application of 20 consecutive pulses, *C_1_* and *C_2_* are coefficients of −0.21 and −0.25, *t* is the time interval between adjacent pulses, *τ_1_* and *τ_2_* are time constant of the SRDP curve and related to be 75.21 and 9.70 µs, respectively, b is the bias value of −0.02. SRDP can be understood in terms of interactions between the adjacent voltage pulses on the back‐migration of the protons. When a single voltage pulse with the amplitude of 0.3 V and width of 10 µs is applied onto the OPM, certain amounts of protons will be pushed back to their original position near the bottom and combine with the sulfonic anions. Upon the removal of the doping energy level and decrease of the built‐in point, the device current decreases consequently. If the time interval between the first and second voltage pulse is long enough, all the protons will reach their new thermodynamic equilibrium state. The lack of interaction between the individual pulse's influences then results in slow decreases in device currents. As the time interval between the adjacent pulses decreases, overlapping of the individual pulse's influences will accelerate the attenuation of the device current. Consequently, an obvious pulse‐interval dependent modulation of device current, which is similar to the SRDP characteristics in biological synapse, is recorded.

The Hebbian spiking‐timing‐dependent plasticity (STDP) can also be realized with the present TPPS organic synapse (Figure 4d). Herein, a pair of identical voltage spikes are applied onto the top electrode sequentially, working as pre‐ and post‐synaptic spikes to modulate device current through self‐coordinated ion migration effect. Each spike is composed of a train of 20 positively biased voltages with the width of 10 µs, interval of 5 µs and amplitudes increasing from 0.01 to 0.2 V in a ramping step of 0.01 V, and a train of 10 negatively biased voltages with the width of 10 µs, interval of 5 µs and amplitudes decreasing from −0.1 to −0.01 V in a declining step of −0.01 V, respectively. When the pre‐spike arrives earlier than the post‐spike and they overlap with each other, the overall effect of their combination is the resulting of a net negative voltage stimulus applied onto the TPPS device. It induces the migration of protons toward the top electrode and causes an increase in the device current. As the time interval between the pre‐ and post‐synaptic spikes decreases, the amplitude of the equivalent negative voltage stimulus increases. Consequently, the weight increase rate changes from 0.25% to 24.43%, as the spike interval decreases from 100 to 5 µs. Contrarily, when the post‐spike arrives earlier, the device current decreases. The synaptic weight decreasing rate of −0.23% to −30.34% is demonstrated for spike intervals of 100 to 5 µs. The STDP characteristics follows the formula below:

(3) $$\Delta \omega \left(t_{\mathrm{Pre}},t_{\mathrm{Post}}\right)=\mathrm{Aexp}\left(-\Delta t_{\mathrm{Post}-\mathrm{Pre}}/\tau \right)+c$$

where *A* is a coefficient of 0.36 for potentiation and −0.44 for depression, Δ*t_Post‐Pre_* represents the time interval between pre‐ and post‐synaptic spikes, *τ* is a time constant of 55.15 for potentiation and −46.84 for depression, and *c* is the bias value of −0.06 for potentiation and 0.05 for depression. The power consumptions for both the SRDP and STDP characteristics vary between 0.66 and 0.82 pW.

Furthermore, endurance test of LTD and LTP was conducted by modulating device currents using multiple voltage pulse trains. Each voltage pulse train consists of 20 consecutive voltage pulses with the amplitude of 0.3 V, width of 10 µs and interval of 24 µs, and subsequently 4 voltage pulses with the amplitude of −0.3 V, width of 10 µs and interval of 5 µs. As shown in Figure 4e,e‐1, the 20 positive voltage pulses can decrease the device current continuously from 82.12 to 66.38 pA, emulating the LTD characteristics of biological synapses. On the other hand, the 4 negative voltage pulses can increase the device current gradually from 68.12 to 82.14 pA, therefore resembling the synaptic LTP behaviors. More importantly, the 100 continuous endurance cycles exhibit tiny average variation of 0.14% and 0.30% in the LTD and LTP branches, respectively, which are calculated according Equation (1) with *δ* and *µ* being the standard deviation and average value of the synapse weights (or device currents). Therefore, the aforementioned ultra‐low power and robust spike‐related plasticities make the present TPPS based OPM devices promising candidates for the implementation of high energy‐efficient and reliable artificial synapses.

## 3 Conclusion

In summary, we design a novel strategy of utilizing proton‐reservoir type organic molecules to construct artificial synapses that can overcome the long‐lasting challenge of achieving ultra‐low power operation and nonvolatile memory simultaneously. Organic molecules TPPS, containing both proton donating sulfonic acid and proton accepting amino groups, are synthesized and used to fabricate memristor devices with the electrode linewidths of 10 µm, 5 µm, 1 µm and 100 nm, respectively. Upon the occurrence of proton migration and self‐coordinated doping, the introduction of intermediate energy levels into the molecular bandgap can effectively and nonvolatilly tune the charge carrier transport properties of TPPS film. Compromising the overall influence of the built‐in potential established across the organic film and the applied external electric field, 64‐state modulation of conductances with approaching‐zero modulating power consumption of 16.25 pW to 2.06 nW, reading power consumption of 6.5 fW to 0.83 pW, as well as retention over 30 min, is demonstrated in Au/TPPS/Au cross‐point structure devices. Through current profile analysis, PFM and in situ electrochemical fluorescence measurements, we ascribe the superior conductance modulation characteristics observed in TPPS devices to sequential proton migration and related interfacial switching phenomena. Finally, an artificial synapse with ultra‐low power SRDP, STDP and robust endurance performance is showcased by the present TPPS‐based OPM device, exhibiting promising potential to develop high‐performance molecular neuromorphic systems. Considering the future challenges of this area, since such intelligent hardware usually relies on large scale array of synaptic devices to achieve high throughput for practical data‐centric edge computing applications, more efforts should be devoted to further optimizing the fabricating and encapsulating techniques for the high‐yield production of organic electronic chips. In addition, the integration of organic electronic chips with the silicon‐based input/output circuits and peripheral digital circuits, as well as the advanced neuromorphic algorithms used to handle complex cognition tasks, should also be attended in the future to implement fully functioning molecular neuromorphic systems.

## 4 Experimental Section

### TPPS Synthesis

TPPS molecules were synthesized via direct sulfonation of TPP, as illustrated in the left panel of Figure 1c. 5.0 g (8.13 mmol) TPP was first added into a 100 mL empty flask. Then, 50 mL concentrated H_2_SO_4_ was added into the flask to dissolve TPP. The mixture was kept at 90 °C for 6 h to achieve a complete sulfonation reaction between the reagents. Subsequently, the mixture was poured into 1 L ice water, while the crude product was precipitated by centrifugation at 24 000 rpm to give green solids. These solids were scratched from the centrifugal tube, redispersed in ice water and centrifugated at 24 000 rpm again to collect the final product. (6.9 g, 90.6%).

### OPM Fabrication and Characterization

The electrical performance of TPPS was assessed in OPM devices with the structure of Au/TPPS/Au and electrode linewidth of 10 µm, 5 µm, 1 µm and 100 nm, respectively. The devices were fabricated through electron beam lithography (EBL) technique and a lift‐off approach on a SiO_2_/Si substrate. The bottom electrode was patterned by EBL with a Vistec EBPG‐5200^+^ Electron‐beam lithography system and E‐beam evaporation of a 50 nm Au layer on top of a 10 nm Ti adhesion layer with a Denton Electron Beam Evaporator.

The fabrication process begins by spin‐coating a PMMA (polymethyl methacrylate) photoresist layer onto a silicon substrate. The coated substrate was then heated at 150 °C for 2 min to allow the photoresist to solidify. Electron‐beam with the exposure dose of 580 µC cm^−2^and exposure time of 10 s per matrix step was then applied onto the selected area of the photoresist layer in a scanning manner to define the pre‐designed hammer shape patterns. Afterward, the developed photoresist layer was subjected to develop and fixation processes using the mixture of methylisobutylketone (MIBK) and isopropanol (IPA) in a 1:3 volume ratio and IPA as solvents, respectively. These processes removed the E‐beam exposed areas of the photoresist and left behind the pre‐designed patterns. Then, an Au/Ti layer was deposited onto the patterned photoresist by E‐beam evaporation. Finally, acetone was employed as the lift‐off solvent to remove the unpatterned areas of the photoresist and the Au/Ti layer deposited on these regions, resulting in the formation of bottom electrode with the pre‐designed hammer shape on the SiO_2_/Si substrate. As shown in Figure 2a, the square‐shape probing pads of the bottom electrodes had a dimension of 60 µm, while the lengths of the connected electrodes strips were 80 µm. The widths of the electrode strips varied between 10 µm and 100 nm. In the second stage, the above‐prepared sample was put into an oxygen plasma treatment oven for 10 min under oxygen atmosphere to make the sample surface hydrophilic. Afterward, 100 µL aqueous solution of 5 mg mL^−1^ TPPS was spin‐coated onto the bottom electrodes at 4000 rpm for 60 s. The samples were then dried at 80 °C in vacuum for 6 h. Finally, the top electrode consisting of 10 nm Ti and 50 nm Au were patterned and deposited using e‐beam lithography, e‐beam evaporation, and lift‐off methods similarly. Therefore, Au/TPPS/Au OPM devices were formed at the cross points of the orthogonally aligned top and bottom electrodes. The cross‐sectional microscopic image of the Au/TPPS/Au devices is shown in Figure S10 (Supporting Information). All electrical measurements of the devices in this work were performed on a Keithley 4200A semiconductor parameter analyzer equipped with a pulse‐measuring unit. Atomic force microscopy (AFM), C‐AFM, and PFM measurements were performed on an FastScan Bio (Bruker Co., American) microscope to monitor the surface morphology, local conduction and polarization behaviors of the TPPS thin film deposited on the Au coated SiO_2_/Si substrate. During C‐AFM and PFM measurements, a conducting cantilever coated with Pt was used as movable and grounded top electrode to form a Pt/TPPS/Au structure, while the voltage pulses were applied through the universal bottom silver electrode to the TPPS thin film.

## Conflict of Interest

The authors declare no conflict of interest.

## Supporting information

Supporting Information

## Data Availability

The data that support the findings of this study are available from the corresponding author upon reasonable request.
