# Supplementary material for: Approaching the Zero‐Power Operating Limit in a Self‐Coordinated Organic Protonic Synapse
Source: Adv Sci (Weinh). 2023 Oct 23;10(34):2305075. doi: 10.1002/advs.202305075 (PMC10700229; doi:10.1002/advs.202305075)
Supplement: Supplementary file 1 — Supporting Information [file ADVS-10-2305075-s001.pdf]

ADVANCED SCIENCE
Open Access

Supporting Information

for *Adv. Sci.*, DOI 10.1002/advs.202305075

Approaching the Zero-Power Operating Limit in a Self-Coordinated Organic Protonic Synapse

Shuzhi Liu, Zhilong He, Bin Zhang, Xiaolong Zhong, Bingjie Guo, Weilin Chen, Hongxiao Duan, Yi Tong, Haidong He, Yu Chen and Gang Liu**

Approaching the Zero-Power Operating Limit in A Self-Coordinated Organic Protonic Synapse

Shuzhi Liu[1,3], Zhilong He[1], Bin Zhang[2], Xiaolong Zhong[3], Bingjie Guo[1], Weilin Chen[3], Hongxiao Duan[3], Yi Tong[4], Haidong He[5], Yu Chen[2*], Gang Liu[1,3*]

S. Liu, Z. He, B. Guo, G. Liu

School of Chemistry and Chemical Engineering, Shanghai Jiao Tong University, Shanghai 200240, China.

E-mail： gang.liu@sjtu.edu.cn (Prof. Gang Liu)

B. Zhang, Y. Chen

School of Chemistry and Molecular Engineering, East China University of Science and Technology, Shanghai 200237, China.

E-mail： 3148704620@qq.com (Prof. Yu Chen)

S. Liu, X. Zhong, W. Chen, H. Duan, G. Liu

Department of Micro/Nano Electronics, School of Electronic Information and Electrical Engineering, Shanghai Jiao Tong University, Shanghai 200240, China.

Y. Tong

Suzhou Laboratory, Suzhou 215000, China.

H. He

Minhang Hospital, Fudan University, 170 Xinsong Road, Shanghai 201199, China.

Table of Contents

Section 1. Disadvantages of charge transfer and conductive filament models

Section 2. Reagents and characterization instruments for TPPS

Section 3. Composition characterization of TPPS

Section 4. Optical properties and energy band gap of TPPS film

Section 5. Structural properties of TPPS film

Section 6. Morphological and local conduction properties of TPPS film

Section 7. Compatibility of TPPS with device fabrication process

Section 8. Electrical performance of Au/TPPS/Au OPM devices

Section 9. Correlation between proton migration, self-coordination and conductance modulation of Au/TPPS/Au OPM devices

Section 10. Experimental setup for *in-situ* electrochemical fluorescence measurements

Section 1. Disadvantages of charge transfer and conductive filament models

As mentioned in the main text, ultra-low power and nonvolatile conductance modulation is a natural paradox for organic neuromorphic devices working on either charge transfer (CT) or metallic conductive filament (CF) mechanisms. Although the small effective mass and large charge-to-mass ratio of electrons usually lead to strong electrostatic driving force, fast response speed and low-power modulation characteristics in CT based processes, they also encounter easy backward relaxation of spontaneous electron-hole recombination and therefore short life time problems (**Figure S1a**). On the other hand, the relatively large volume and

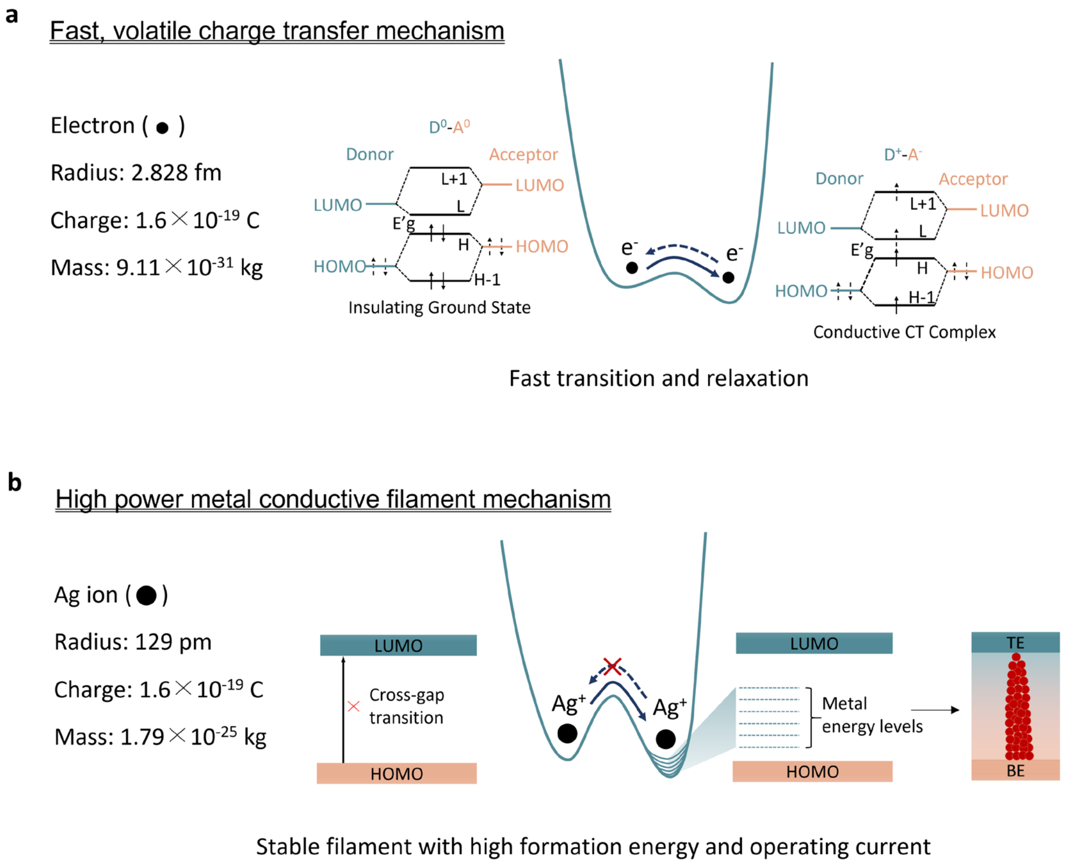

Figure S1. Energy band diagrams and working mechanisms of organic neuromorphic devices based on (a) charge transfer and (b) metal conductive filament models.

mass of metal cations can guarantee long-term stability of thick conductive filament formed upon ion migration and redox reaction. The high electric field used to inject abundant metal ions from electrode into organic switching layer, as well as the excellent metallic conduction of the as-formed filament that carries high current during device operation, nevertheless, give rise to significant power consumption that is obviously undesired for large scale integration and applications (**Figure S1b**).

Section 2. Reagents and characterization instruments for TPPS

5,10,15,20-tetraphenylporphyrin (TPP) was purchased from Bide Pharmatech Ltd. and used without further purification. Concentrated sulphuric acid (H_2SO_4) was purchased from Sinopharm Group Co. Ltd. and used without further purification. The ^1H nuclear magnetic resonance (NMR) spectra were recorded on a Bruker AVANCE III HD 500 spectrometer at 500 MHz in deuterium substituted water with tetramethylsilane (TMS) as reference for the chemical shifts. The matrix-assisted laser desorption/ionization time of flight mass spectrometry (MALDI TOF-MS) was measured on Bruker autoflex speed TOF/TOF. The Fourier transformed infrared (FT-IR) spectrometry was conducted on Thermo Fisher Nicolet 6700. The cyclic voltammetry (CV) measurements were performed on a model CHI 650D electrochemical workstation with tetrabutyl ammonium hexafluorophosphate (0.10 M) in acetonitrile as the supporting electrolyte, a platinum disk as the working electrode, an Ag/AgCl electrode as the reference electrode, and a Pt wire as the counter electrode, respectively. Tetrabutyl ammonium hexafluorophosphate was recrystallized using chloroform to remove the low-content impurity before using. The UV-visible absorption spectral measurement was conducted on PerkinElmer Lambda 750S. The fluorescence spectrum was measured on Renishaw inVia Qontor with fluorescence mode. Grazing incidence wide angle X-ray scattering (GIWAXS) experiments were performed using a Xenocs Xeuss 3.0 beamline at the Vacuum Interconnected Nanotech Workstation (NANO-X) of Suzhou Institute of Nano-Tech and Nano-Bionics, Chinese Academy of Sciences. Xeuss 3.0 (GI-)SAXS/WAXS/USAXS beamline was equipped with dual X-ray sources, an Excillum high flux gallium metal jet source and a Xenocs GeniX^{3D} micro-focus copper source. An X-ray wavelength of $\lambda = 1.3414$ Å and a beam size of (0.9×0.9) mm^2 were

employed for the measurements. The scattered intensities were collected using a Dectris EIGER2 R 1M detector at a sample to detector distance of 130 mm, with an instrument resolution of ~0.02 $Å^{-1}$. GIWAXS data were collected with an exposure time of 1200 s and X-ray incident angle of 0.2°. Atomic force microscopy (AFM), conductive atomic force microscopy (C-AFM) and piezo force microscopy (PFM) measurements were performed on an FastScan Bio (Bruker Co., American) microscope to monitor the surface morphology, local conduction and polarization characteristics of the TPPS film deposited on Au coated SiO_2/Si substrate.

Section 3. Composition characterization of TPPS

^{1}H NMR (DMSO-d$_6$, 400 MHz) δ (ppm): 8.80 (s, 8H), 8.70 (br, 8H), 8.34 (br, 8H) (**Figure S2**). It should be noted that the chemical shift of δ=4.07 can be attributed to the cation of H_3O^+ produced from the coordination between H_2O molecule and proton ($H_2O + H^+ \rightarrow H_3O^+$), which exchanges hydrogen element with deuterated DMSO and thus results in an obvious shift to the low field. MALDI TOF-MS ($C_{44}H_{30}N_4O_{12}S_4$) m/z: calcd. 934.989, found: 935.03 (**Figure S3a**). According to the FT-IR spectrum of TPPS, the peak at *ca* 1228 cm^{-1}, 1192 cm^{-1} and 1122 cm^{-1} can be ascribed to the SO_3^- vibrations, which indicates a successful synthesis with sulfonic acid group (**Figure S3b**).

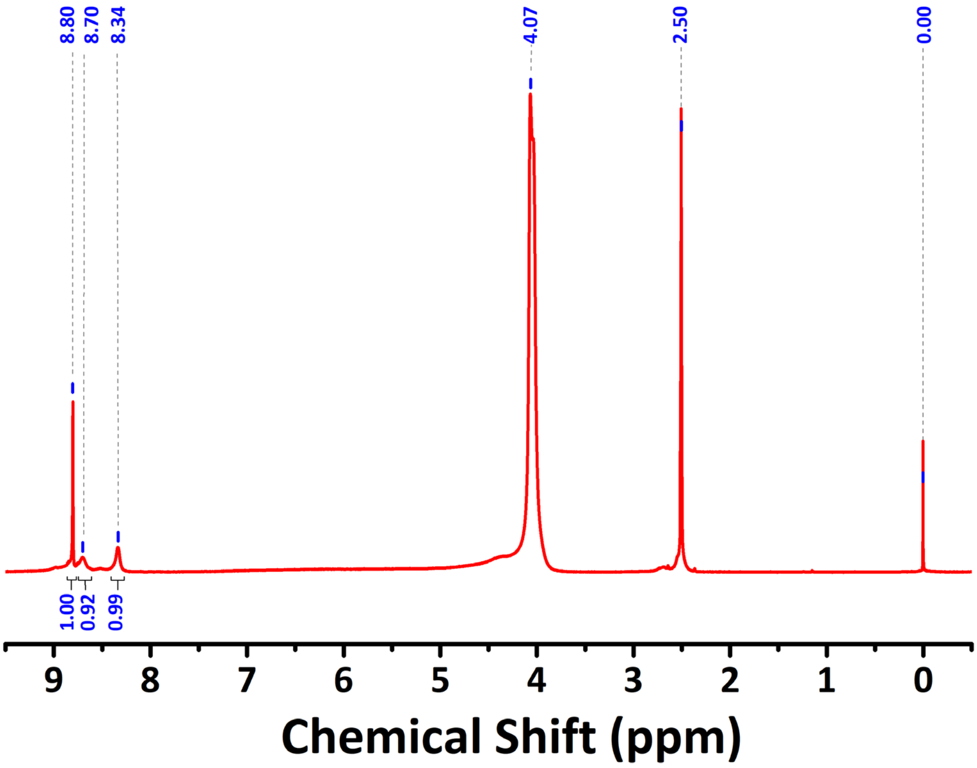

Figure S2. ^{1}H NMR spectrum of TPPS.

Section 4. Optical properties and energy band gap of TPPS film

Benefiting from these characterizations aforementioned to ensure a success synthesis of TPPS molecule, we subsequently evaluate its optical and structural properties in the condensed state. A thin film of TPPS was formed on quartz substrate by spin-coating. Before spin-coating, the quartz substrate was pre-treated with oxygen plasma for 10 min to make the surface hydrophilic. As shown in **Figure S4**, the TPPS film shows intrinsic absorption peaks at the wavelengths of 366, 428 and 490 nm, while the absorption peaks at 633 and 702 nm are ascribed to the π-π stacking of the planar molecules in the TPPS film.[1] The latter absorptions at the longer wave-lengths of 633 and 702 nm indicate that TPPS film may have the ordered microstructure in solid state. When the TPPS film is excited by green light with the wavelength of 532 nm, a wide emission peak centered at 717 nm can be observed. The difference in the peak positions of fluorescence emission (717 nm) and maximum visible absorption (702 nm) gives rise to a small Stokes shift of 15 nm, again suggesting good crystallinity of the TPPS film that suppresses the vibrational relaxation of the activated-state molecules.

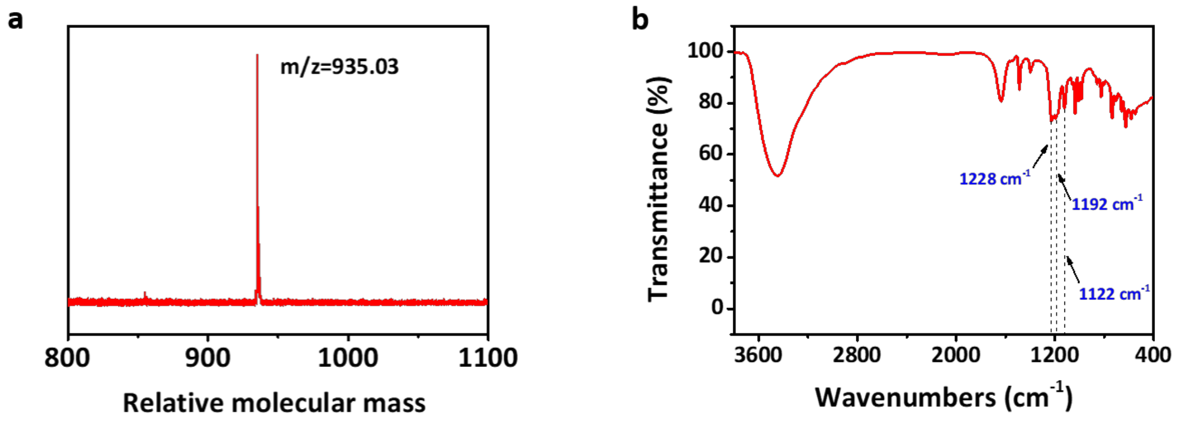

Figure S3. (a) MALDI TOF-MS and (b) FT-IR spectra of TPPS.

Figure S5a converts the UV-visible absorption spectrum of the TPPS film into a Tauc plot, in which $(\alpha h\nu)^n$ is displayed as a function of $h\nu$ according to the equation $(\alpha h\nu)^n = B(h\nu - E_g)$. With this plot, the energy band gap of TPPS can be calculated as the intersection of the first absorption peak's tangent with the X-axis, where α is the linear absorption coefficient of TPPS film, h=6.63×10^{-34} J·s is the Planck's constant, ν is the photon frequency and B is a material related constant. α equals 2.303×A/d, where A and d are the absorption intensity and thickness of the TPPS film, respectively. n, with the value of 2 or 1/2, is related with electron hopping behavior and used to calculate the direct band gap or indirect band gap, respectively. Since most organic semiconductor materials have indirect band gaps,[2] herein we employ n=1/2 to derive the above function and plot in **Figure S5a**. Consequently, the energy band gap of TPPS film is read as the intersection of first absorption peak's tangent with the X-axis, which is 1.64 eV.

On the other hand, ultraviolet photoelectron spectroscopy (UPS) measurement is used to ascertain the the Fermi level (E_F) and the valence band maximum (E_V) in comparison to vacuum

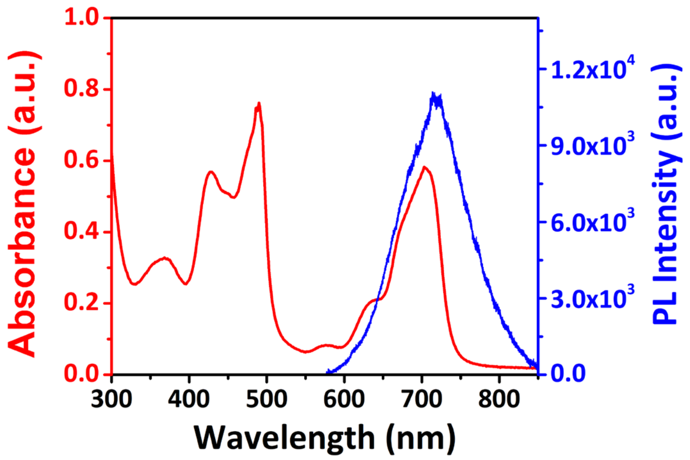

Figure S4. UV-visible absorbance and fluorescence spectra of TPPS thin films.

level (E_{VAC}) for TPPS film.[3] In order for a photoelectron to escape from the surface of a sample and to be detected, it must possess enough energy to overcome the sum of the binding energy of its initial level with respect to the the work function of the sample (Φ) and Fermi level, which is equal to the difference between the incident photon energy (21.22 eV) and the binding energy of the secondary electron cut-off. The secondary electron cut-off, which represents photoelectrons with zero kinetic energy (E_k) when they escape from the sample surface, occurs at a fixed incident photon energy of 21.22 eV. As shown in the upper panel of **Figure S5b**, the cut-off binding energy is determined to be 16.71 eV, based on the intersection of the linear portion of the spectrum and the baseline. As such, the work function of this sample can be calculated as $\Phi = 21.22 - 16.71 = 4.51$ eV, indicating that the Fermi level (E_F) is -4.51 eV. The difference between the Fermi level and valence band maximum (E_V) can be determined by finding the intersection of the linear portion of the spectra near the Fermi edge (low binding energy region) with the baseline. As shown in the lower panel of **Figure S5b**, E_F-E_V = 1.20 eV, so the valence band maximum is -5.71 eV. Then, the conduction band minimum (E_C) can be calculated by adding the value of the band gap ($E_C = E_V + Eg$), which is -4.07 eV.

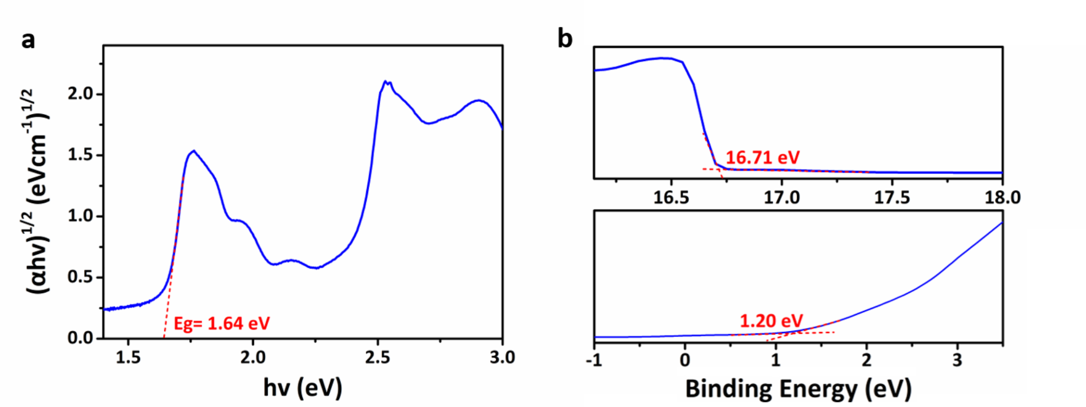

Figure S5. (a) Tauc plot of the TPPS film's UV-visible absorbance spectrum. (b) UPS measurement results of the (top) secondary electron cutoff and (bottom) ionization edge.

Section 5. Structural properties of TPPS film

Grazing incident wide-angle X-ray scattering (GIWAXS) measurement was also conducted to assess the crystallinity of TPPS films. As shown in **Figure S6**, the GIWAXS profile of TPPS film shows a scattering ring at the scattering vector of 1.48 Å, which can be ascribed to the in-plane isotropic alignment of the grains' (001) lattice plane. The minor anisotropic signal observed at the scattering vector of 0.42 Å, on the other hand, is in good agreement with the ordered π-π stacking of the planar TPPS molecules in the out-of-direction.

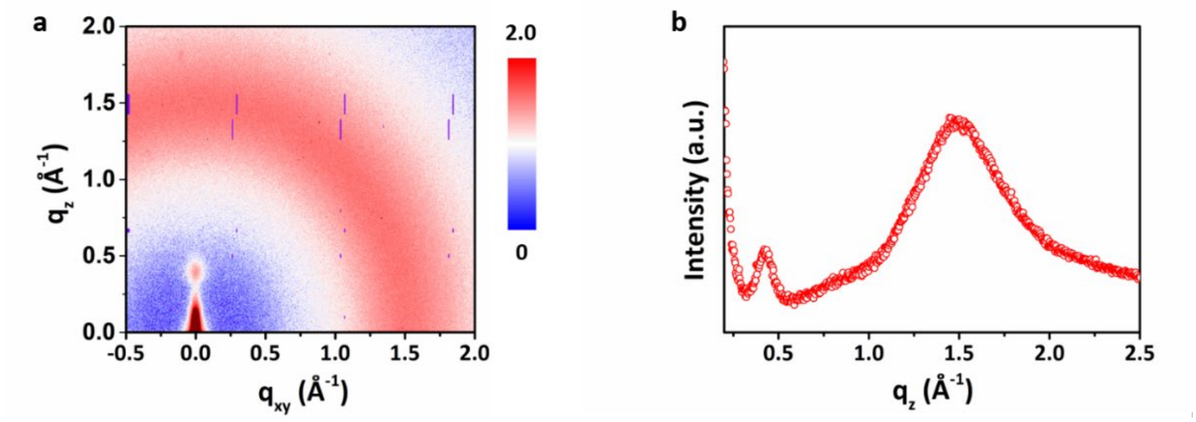

Figure S6. (a) 2D GIWAXS pattern and (b) out-of-plane direction X-ray scattering intensity profile of the TPPS thin film.

Section 6. Morphological and local conduction properties of TPPS film

As depicted in **Figure S7a**, the atomic force microscope (AFM) profile of the TPPS thin film further proves featureless alignment of crystalline grains with a root-mean-square roughness of 1.632 nm. Such uniform distribution of the organic crystals can provide a three-dimensional continuous network for ion migration. Conductive atomic force microscope (C-AFM) scan over the area of 1 μm×1 μm shows a uniform tiny leakage current of 0.09 pA (read at 0.01 V), suggesting that the initial TPPS film is in the high resistance state (**Figure S7b**). When -1.5 V voltage stimuli were applied point-by-point onto the TPPS thin film through C-AFM cantilever, densely distributed local conductive regions with maximum current exceeding 25 pA can be observed (**Figure S7c**). It indicates that the TPPS film has been switched to a high conductance state. Zooming-in observation from 1 μm×1 μm to 0.5 μm×0.5 μm, 0.1 μm×0.1 μm and 50 nm×50 nm scanning areas reveals that the inter-spacing between the adjacent conductive regions can be as small as 10 nm, therefore manifesting the downscaling potential of TPPS-based OPM devices into nanometer range.

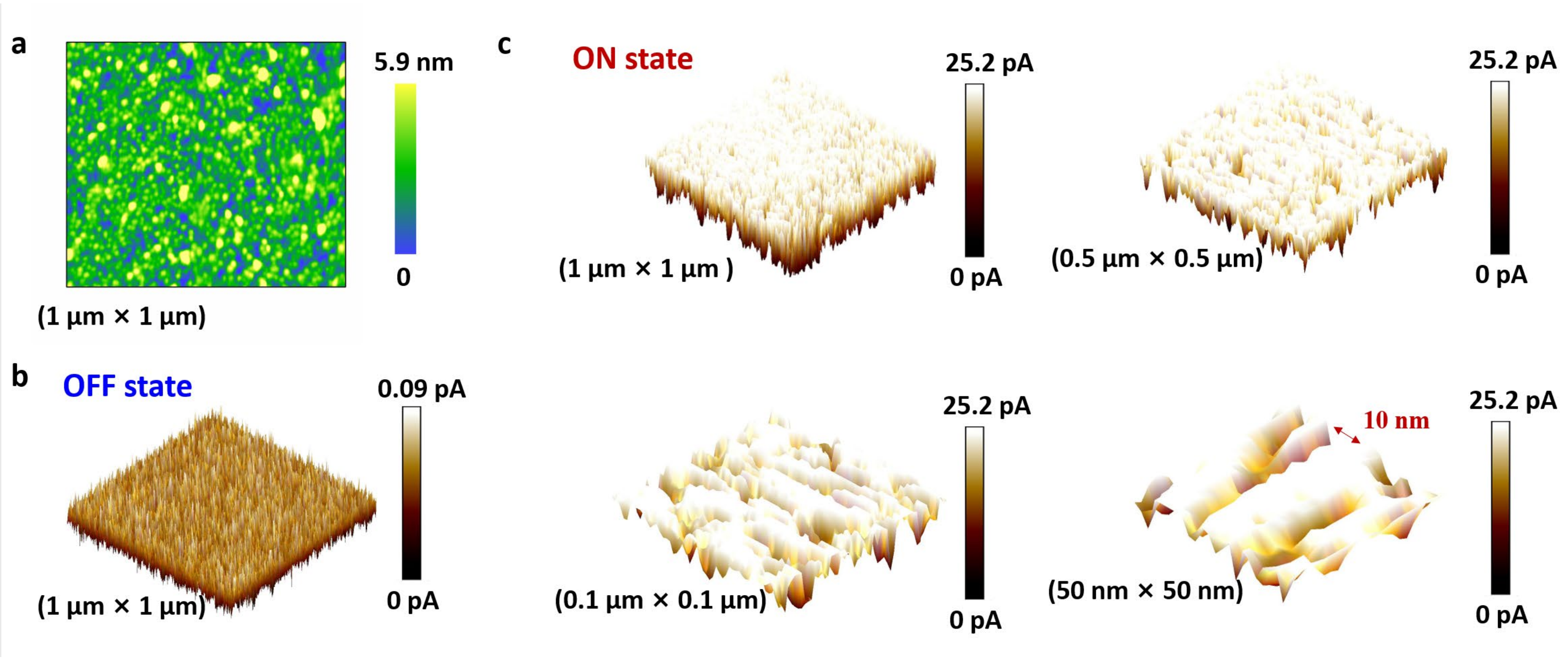

Figure S7. (a) Surface morphology and (b) current profile of the pristine TPPS film scanned by AFM and C-AFM over a 1 µm×1 µm area. (c) Current mapping images of the high conductance state TPPS film over the scanning areas of 1 µm×1 µm, 0.5 µm×0.5 µm, 0.1 µm×0.1 µm and 50 nm×50 nm, respectively.

Section 7. Compatibility of TPPS with device fabrication process

To validate the compatibility of TPPS molecules with the device fabrication process, we firstly conducted solubility tests on the developing, fixing, and stripping solvents of methylisobutyl-ketone (MIBK) and isopropanol (IPA) mixture in a 1:3 volume ratio, isopropanol (IPA), and acetone, respectively. 20 mg powders of TPPS were added into each of the 3 mL above solvents. After resting for 3 days, direct observation was conducted to check whether the TPPS powders were dissolved by these solvents or not. As shown in **Figure S8**, the upper liquid remained colorless while the solids were all precipitated in the bottom of the glass bottle. Upon discarding the liquids and drying the solids in vacuum oven at 60 °C overnight, it was found that the weight of each TPPS sample was in close proximity to 20 mg. These results suggest that TPPS does not dissolve in the solvents used in the e-beam lithography process. It is in good agreement with the "like-dissolves-like rule" in fundamental chemistry, wherein TPPS is highly polar with tethered strong electron withdrawing sulfonic acid groups, while the above solvents carry either low or moderate polarities.

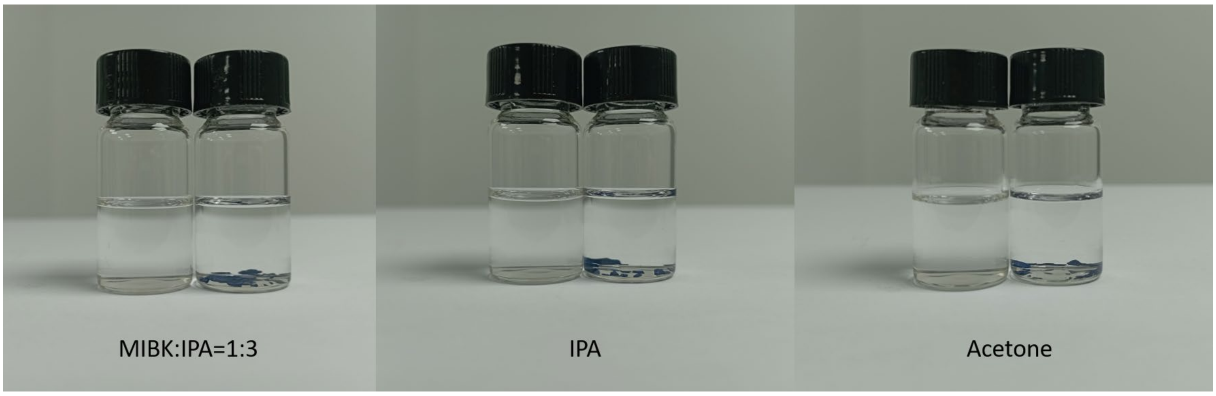

Figure S8. Images of MIBK/IPA, IPA and acetone solvents (left sample in each image) and mixture of TPPS powders with these solvents (right sample in each image), respectively. The images were taken 3 days after adding the TPPS powers into the solvents.

We further employed spectrometry to confirm the stability of TPPS thin film against EBL and other fabrication processes. Herein, TPPS thin films was subjected to electron-beam exposure, as well as being soaked in the above-mentioned development, fixation and lift-off solvents, sequentially. UV-visible absorption, fluorescence and FTIR spectra of the TPPS thin films were recorded before and after being subjected to these device fabrication processes. Please refer to detailed device fabrication process in Methods section of the manuscript, as well as Section S4 for detailed description of spectroscopic measurements. As plotted in **Figure S9**, the lineshapes of the respective spectra almost do not change after being subjected to the device fabrication processes, confirming that the TPPS thin films are stable against the electron-beam lithography and other device fabrication processes.

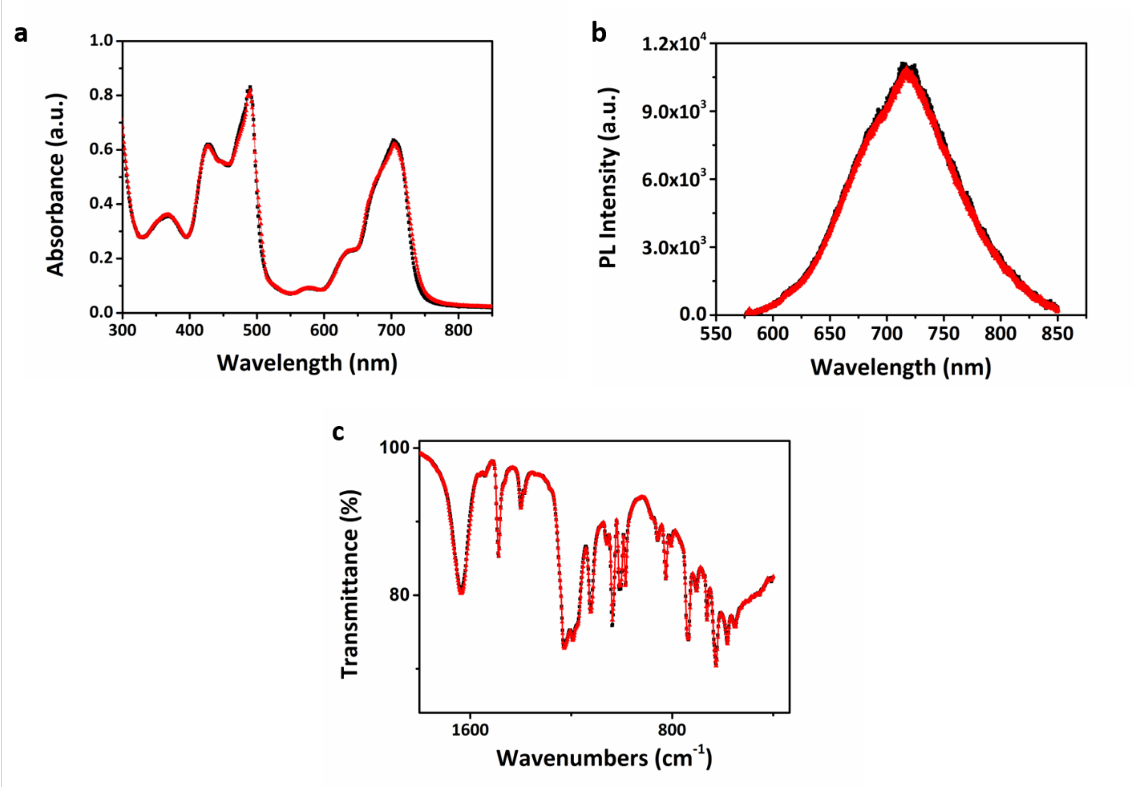

Figure S9. (a) UV-visible absorption, (b) fluorescence and (c) FTIR spectra of the TPPS thin films before (black curves) and after (red curves) being subjected to the device fabrication processes, respectively.

Section 8. Electrical performance of Au/TPPS/Au OPM devices

Figure S10 visualizes the cross-sectional view of the Au/TPPS/Au OPM device. It is clear that the TPPS film spin-coated on the Au electrode coated SiO_2/Si substrate has a thickness of ~ 18 nm.

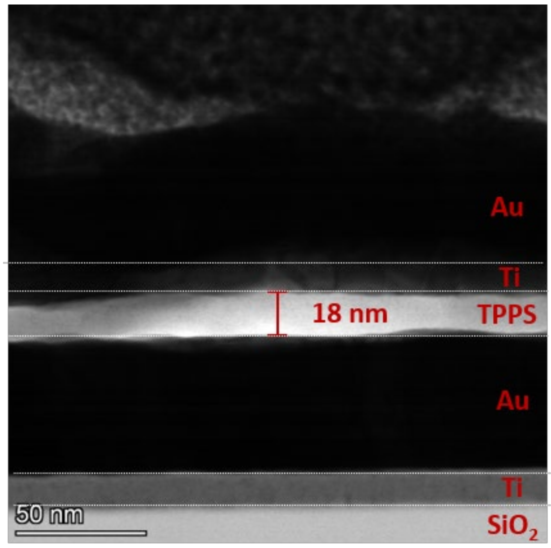

Figure S10. Cross-sectional transmission electron microscope (TEM) image of the Au/TPPS/Au OPM device fabricated on SiO_2/Si substrate.

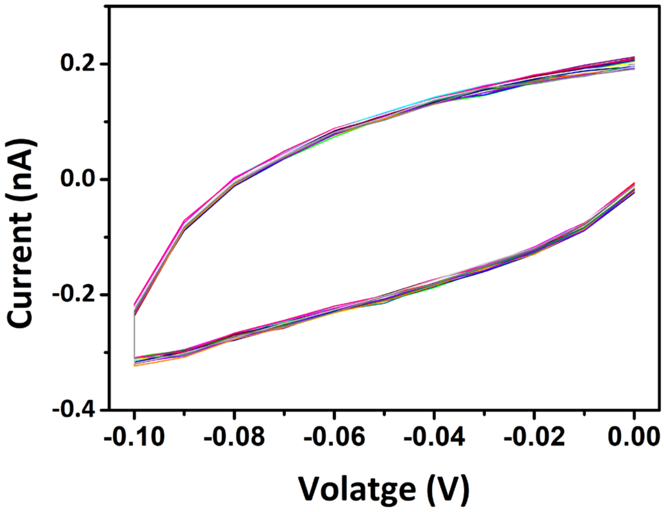

Figure S11. Current-voltage characteristics of 20 Au/TPPS/Au devices exhibiting hysteretic loops.

In order to confirm the validity of the material designing principle in this work, we fabricated 20 TPPS devices with the dimension of 10 μm and tested their electrical characteristics. As plotted in **Figure S11**, all the devices show almost identical hysteresis in the current-voltage characteristics when sweeping between 0 V and -0.1 V. The device-to-device (D2D) uniformity of the conductances of these organic synapses is calculated as 93.49%, according to the following equation

$$\text{Uniformity (\%)} = 1 - (\delta/\mu \times 100\%) \quad (1)$$

where δ and μ are the standard deviation and average values of the 20 devices' conductances read at -0.01 V, respectively. It suggests that the TPPS devices show promising uniformity and reliability.

Figure S12 replots the conductance-voltage characteristics of the TPPS device during sweeping from -0.1 V to -1.17 V, with the data retrieved from **Figure 2c**. As shown, the device conductances switches obviously. Upon the measurements of **Figure 2c**, the OPM is further swept with the stopping voltage increasing from -1.0 V to -5.0 V with a ramping step of -0.1 V. As shown in **Figure S13a**, the device currents read at -0.01 V increase from 0.36 nA to 1.0 nA accordingly. The power consumption of reading the device during this negatively biased modulation period varies from 3.64 pW to 10.06 pW, respectively (**Figure S13b**). The built-in field gradually varies from 0.44 V to 1.22 V (**Figure S13c**). A reserved sweeping from 0 V to 0.8 V consequently recovers the device conductance to the initial state as a reset operation (**Figure S13d**). When successive positively biased dual sweepings with the stopping voltages increasing from 0.1 V to 1.0 V with a ramping step of 0.01 V are performed, the device currents read at 0.01 V decreases from 0.26 nA to 22.51 pA, respectively (**Figure S14a**). The corresponding

reading power consumption varies between 2.61 pW and 0.23 pW, while the built-in potential established across the TPPS film are between 0 V and -0.5 V (**Figure S14b, c**). The conductance evolution during the positive sweeping with the stopping voltages increasing from 0.1 V to 1.0 V is shown in **Figure S15**. Likely, a reverse sweeping from 0 to -0.2 V can erase the device returning to the initial state (**Figure S14d**).

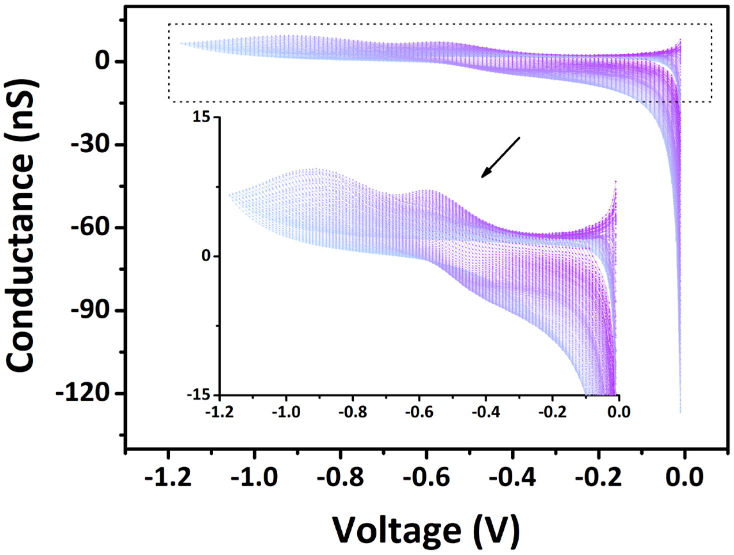

Figure S12. Conductance-voltage characteristics of TPPS device during sweeping between -0.1 V and -1.17 V, with the data retrieved from Fig. 2c.

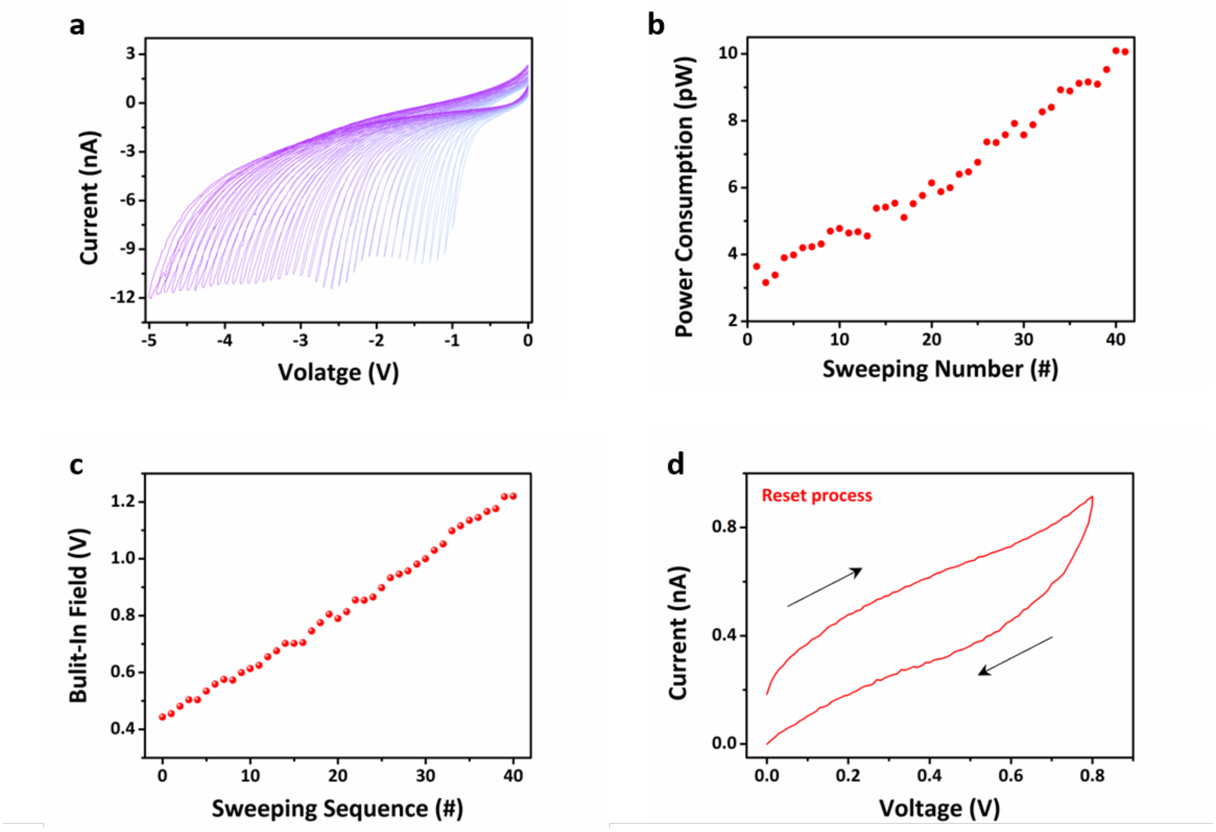

Figure S13. (a) Current-voltage characteristics and (b) reading power consumptions of the Au/TPPS/Au device during the negatively biased voltage sweepings with the stopping voltages varying between -1.0 V and -5.0 V with a ramping step of -0.1 V. (c) Evolution of the built-in potential established across the TPPS film during the negatively biased voltage sweepings shown in (a). (d) Current-voltage characteristics of the device during the positively biased voltage sweeping between 0 V and 0.8 V.

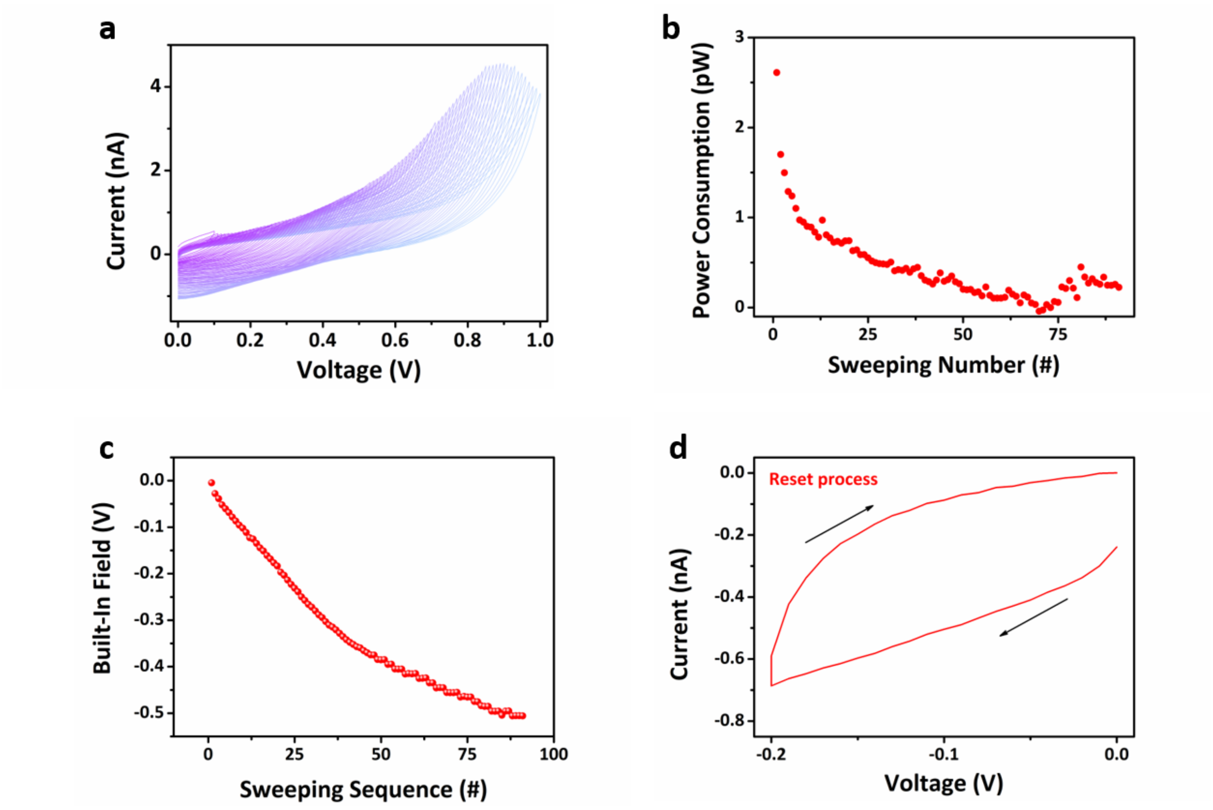

Figure S14. (a) Current-voltage characteristics and (b) reading power consumptions of the device during the positively biased voltage sweepings with the stopping voltages varying between 0.1 V and 1.0 V with a ramping step of 0.01 V. (c) Evolution of the built-in potential established across the TPPS film during the positively biased voltage sweepings shown in (a). (d) Current-voltage characteristics of the device during the negatively biased voltage sweeping between 0 V and -0.2 V.

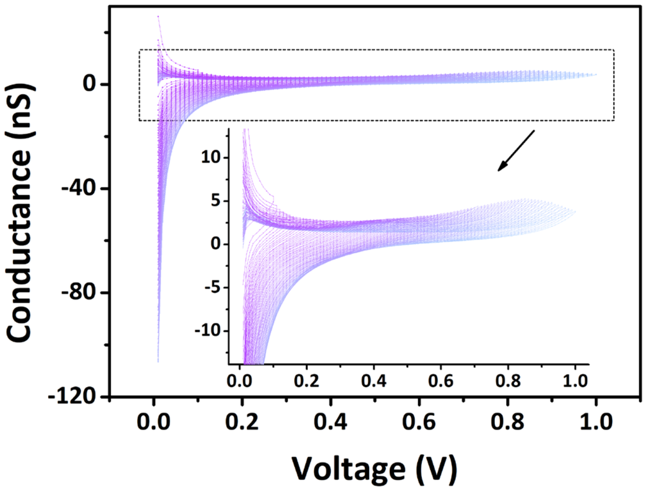

Figure S15. Conductance-voltage characteristics of TPPS device during sweeping between 0.1 V and 1.0 V, with the data retrieved from Figure S14a.

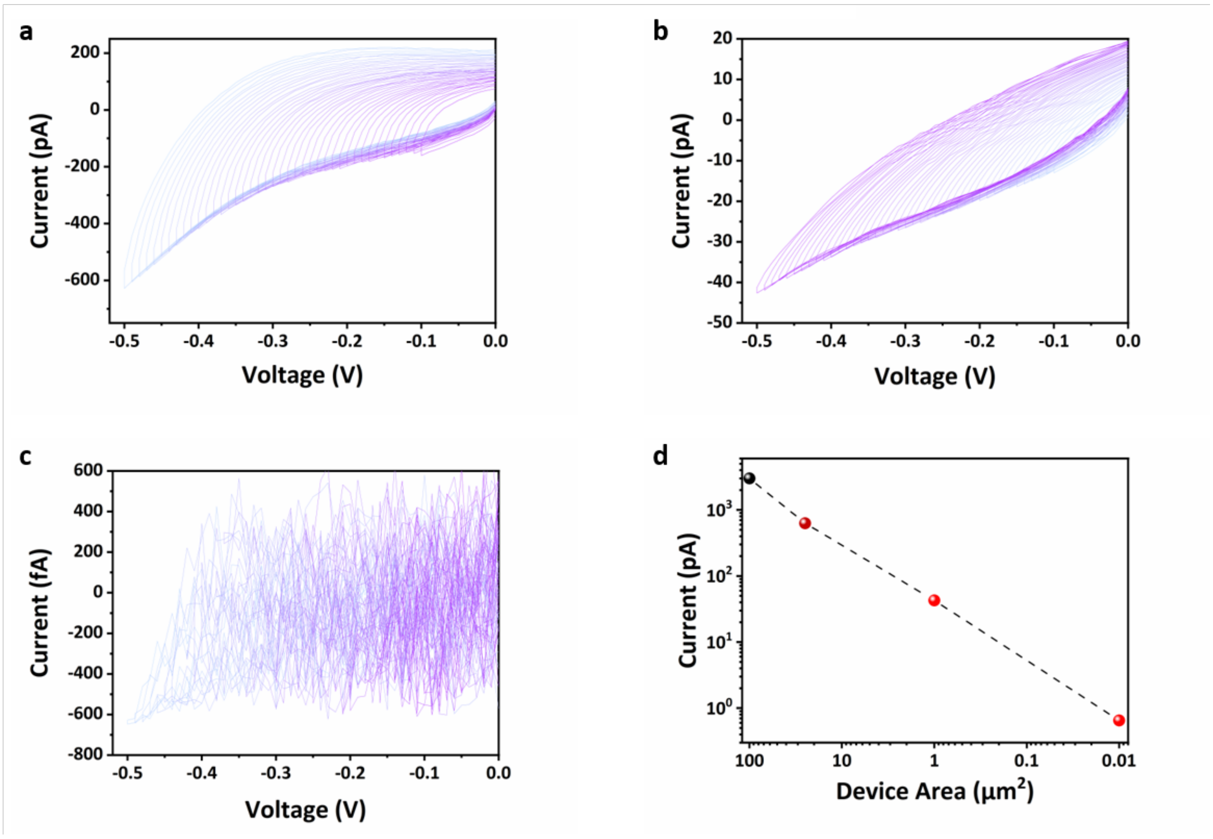

Figure S16. Current-voltage characteristics of the Au/TPPS/Au devices with metal electrode linewidths of (a) 5 µm, (b) 1µm and (c) 100 nm, respectively. All these measurements are performed by negatively biased voltage sweepings with the stopping voltages increasing from -0.1 V to -0.5 V with a ramping step of -0.01 V. (d) Linear dependence of the device current maxima on the metal electrode linewidths.

TPPS based OPM devices with small electrode linewidth of 5 µm, 1µm and 100 nm are also fabricated to assess low-power operation capabilities (Figure 2a). As shown in **Figure S16a** and **16b**, both the 5 µm and 1 µm OPM devices exhibit continuous current modulation during negatively-biased voltage sweepings with the stopping voltages increasing from -0.1 V to -0.5 V with a ramping step of -0.01 V. When read at -0.5 V, the maximum currents of the 5 µm and 1 µm devices are 628.05 pA and 42.62 pA, respectively. When the electrode linewidth further shrinks to 100 nm, the device currents drop dramatically to the detecting limit of the semiconductor parameter analyzer, which is ~ 648.0 fA as read at -0.5 V (**Figure S16c)**. Coincidently,

the current maxima read at -0.5 V for the 10 μm, 5 μm, 1μm and 100 nm devices show a good linear dependence on the device area (**Figure S16d**), suggesting that the conductance tuning in TPPS devices occurs as an interfacial phenomenon.

Section 9. Correlation between proton migration, self-coordination and conductance modulation in Au/TPPS/Au OPM devices

As shown in **Figure S17**, the current-voltage characteristics of the TPPS device can be divided into several current slowly-increasing, current fast-increasing and even negative differential resistance (NDR) regions. The device current increases slowly when swept between 0 V and -0.33 V, which can be ascribed to the proton migration towards the top electrode, their accumulation near the top electrode and establishment of a downward point built-in potential that hinders the charge carrier transport across the organic thin film. When the sweeping voltage exceeds -0.33 V and counterbalances the influence of the built-in potential, a fast increase in the device current can be observed until -0.56 V. When the stopping voltages of the negatively biased sweeps increase from -0.56 V to -0.67 V, the second batch of protons start to migrate

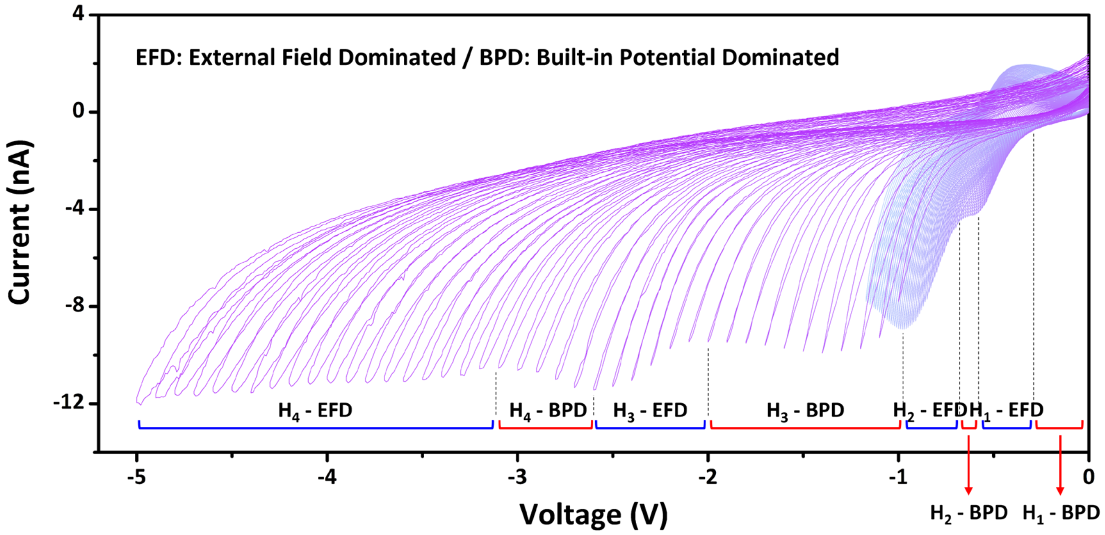

Figure S17. Current-voltage characteristics of the Au/TPPS/Au devices during negatively biased voltage sweepings between 0 V and -5.0 V. Regions showing slow and fast current-increasng behaviors, as well as negative differential resistance (NDR) effect, can be observed.

towards the top electrode and establish a stronger built-in potential. As result, the device current again increases slow in this period. When the stopping voltage further extends to -0.92 V, the influence of the built-in potential is overcome by the external electric field, the device current increases at a faster rate again. Similar phenomena occur when the stopping voltages of the sweeps increase to -5.0 V, when the third and fourth (last) batches of the protons migrate and get accumulated at the top electrode/organic film interface.

In order to better understand the relationship between proton migration, self-coordinated doping and conductance modulation characteristics of the TPPS based OPM devices, molecular simulation was conducted to visualize the evolution of TPPS molecule geometry during proton migration procedure, using the Gaussian 09 program package and the density functional theory (DFT)[4]. The B3LYP hybrid function and an all-electron Pople double zeta basis set 6-31G(d) were adopted for geometry optimization in the ground state. For better comparison between different charged states of TPPS molecule, all states for geometry optimization and excited-state calculation are in singlet and close shell. All geometry optimization was done in the gas phase. The evolution of the molecular geometry and dihedral angles between the sulfonic groups and planar porphyrin framework, along with the excitation and migration of protons from the TPPS molecule, is summarized in **Figure S18**.

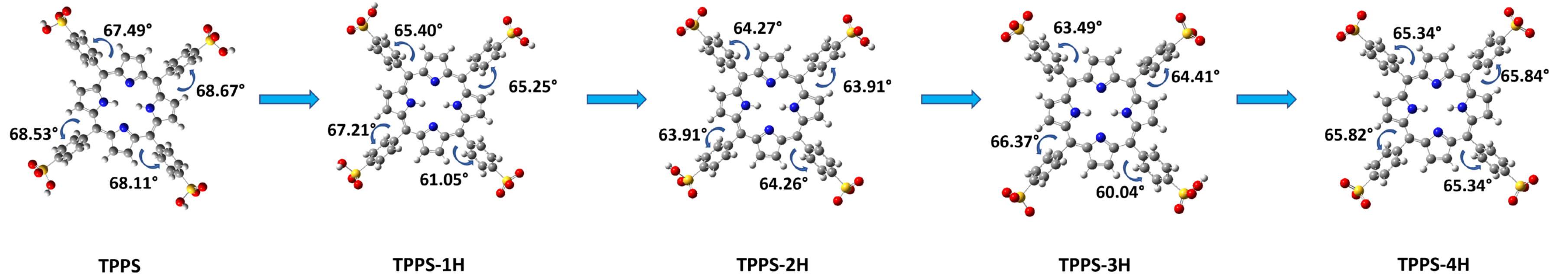

Figure S18. Evolution of the molecular geometry and dihedral angles between the sulfonic groups and planar porphyrin framework, along with the excitation and migration of protons from the TPPS molecule.

Section 10. Experimental setup for *in-situ* electrochemical fluorescence measurements

In order to conduct the *in-situ* electrochemical fluorescence and current response measurements, a lateral structured Au/TPPS/Au device was fabricated on optical-grade quartz substrate (**Figure S19**). Before fabrication, the 2.5 cm×2.5 cm quartz substrate was treated in an oxygen plasma oven for 10 mins under oxygen atmosphere to make the sample surface hydrophilic. Afterwards, 100 μL aqueous solution of 5 mg/mL TPPS was spin-coated onto the substrate at 4000 rpm for 60 s. The sample was then dried at 80 °C in vacuum for 6 h to form the TPPS thin film. Finally, a piece of stainless-steel stripe with the width of 0.5 cm was placed on top of the middle part of the TPPS thin film, servicing as a shadow mask for the deposition of parallel Au electrodes. As such, the lateral structure Au/TPPS/Au device has an active area of 0.5 cm×2.5 cm. The as-fabricated sample was then put onto the sample stage of a Renishaw inVia Qontor system, and connected to external DC source through metal wires. Electrochemical fluorescence spectra of the Au/TPPS/Au device were then recorded on the exposed TPPS layer near the anode, with voltage stimuli applied by the external DC resource through the parallel electrodes. The dc current-voltage characteristics of the lateral Au/TPPS/Au device was recorded on probe station using a Keithley 4200A semiconductor parameter analyzer.

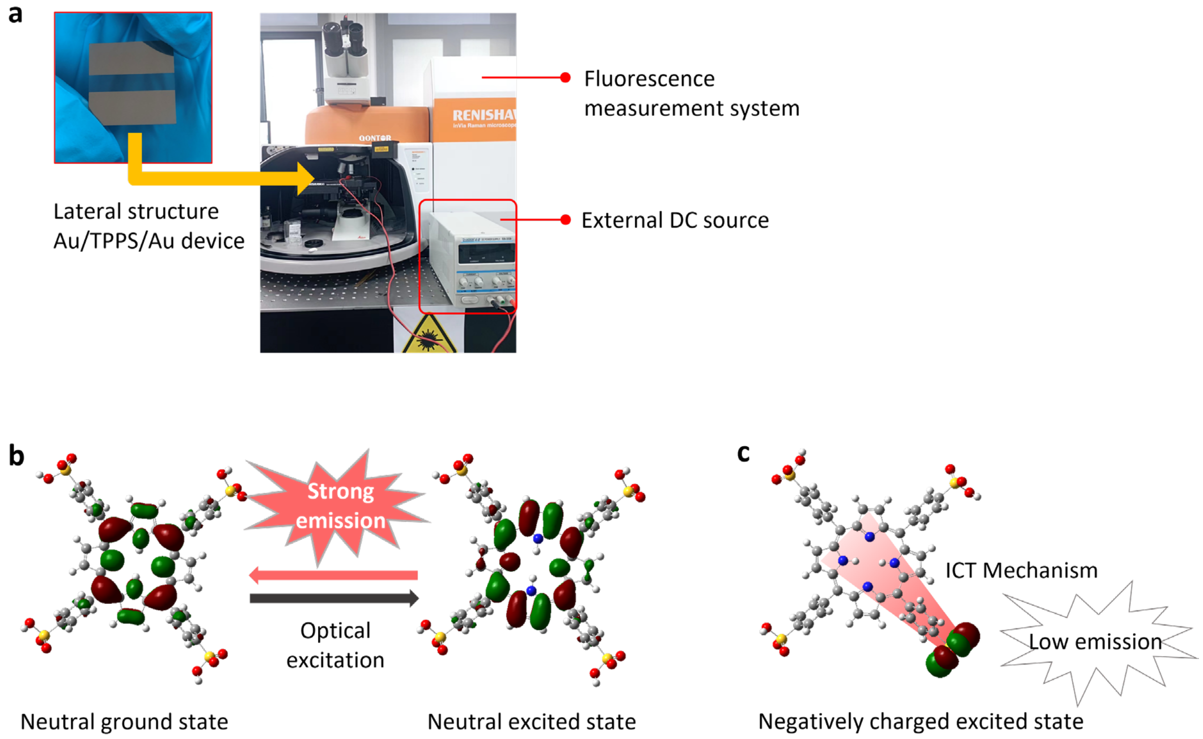

Figure S19. (a) Lateral structured Au/TPPS/Au device and experimental setup for *in-situ* electrochemical fluorescence measurements. (b) Distributions of molecular orbitals of TPPS in its neutral ground state and excited state. As shown, electron transfer from the neutral excited state TPPS to neutral ground state TPPS will emit strong fluorescence. (c) Distribution of molecular orbital of TPPS in its negatively charged excited state. The shift of electron cloud from the central porphyrin skeleton to the peripheral sulfonic moiety upon proton migration and intramolecular charge transfer will attenuate the intensity of TPPS fluorescence.

References

1. L. Liu, Y. Li, and M. Liu. *J. Phys. Chem. C.* **2008**, 112, 4861-4866.

2. J. Liu, W. Zhou, J. Liu, I. Howard, G. Kilibarda, S. Schlabach, D Coupry, M Addicoat, S. Yoneda, Y. Tsutsui, T. Sakurai, S. Seki, Z. Wang, P Lindemann, E Redel, T. Heine, C. Wöll. *Angew. Chem. Int. Edit.* **2015**, 54, 7441-7445.

3. C. Chuang, P. Brown, V. Bulović & M. Bawendi. *Nature Mater*. **2014**, 13, 796-801.

4. Frisch M. J. et al. *GAUSSIAN 09*, revision A.02 (Gaussian, Inc., Wallingford, CT, 2009).
